# Artificial ants deposit pheromone to search for regulatory DNA elements

**DOI:** 10.1186/1471-2164-7-221

**Published:** 2006-08-30

**Authors:** Yunlong Liu, Hiroki Yokota

**Affiliations:** 1 Division of Biostatistics, Department of Medicine, Center for Computational Biology and Bioinformatics, Indiana University – Purdue University Indianapolis, Indianapolis, IN 46202, USA; 2 Department of Biomedical Engineering, Indiana University – Purdue University Indianapolis, Indianapolis, IN 46202, USA

## Abstract

**Background:**

Identification of transcription-factor binding motifs (DNA sequences) can be formulated as a combinatorial problem, where an efficient algorithm is indispensable to predict the role of multiple binding motifs. An ant algorithm is a biology-inspired computational technique, through which a combinatorial problem is solved by mimicking the behavior of social insects such as ants. We developed a unique version of ant algorithms to select a set of binding motifs by considering a potential contribution of each of all random DNA sequences of 4- to 7-bp in length.

**Results:**

Human chondrogenesis was used as a model system. The results revealed that the ant algorithm was able to identify biologically known binding motifs in chondrogenesis such as AP-1, NFκB, and sox9. Some of the predicted motifs were identical to those previously derived with the genetic algorithm. Unlike the genetic algorithm, however, the ant algorithm was able to evaluate a contribution of individual binding motifs as a spectrum of distributed information and predict core consensus motifs from a wider DNA pool.

**Conclusion:**

The ant algorithm offers an efficient, reproducible procedure to predict a role of individual transcription-factor binding motifs using a unique definition of artificial ants.

## Background

To extract biologically meaningful information from a large amount of gene expression data and genomic information is one of the most challenging tasks in the post Human Genome Project era [1-3]. Since transcriptional processes are regulated combinatorially by multiple regulatory elements, computational modeling is inevitable and an efficient algorithm capable of solving combinatorial optimization problems is desirable [4]. Swarm intelligence is a computational technique that mimics the collective behavior of social insects such as ants and bees [5-7]. Although there is no centralized module that dictates the behavior of individuals, local interactions cause a global optimization pattern to emerge [8-10]. Algorithms based on swarm intelligence have been applied successfully to a large number of hard discrete optimization problems including traveling salesman, quadratic assignment and routing in telecommunications networks [6,8].

**Figure 1:**
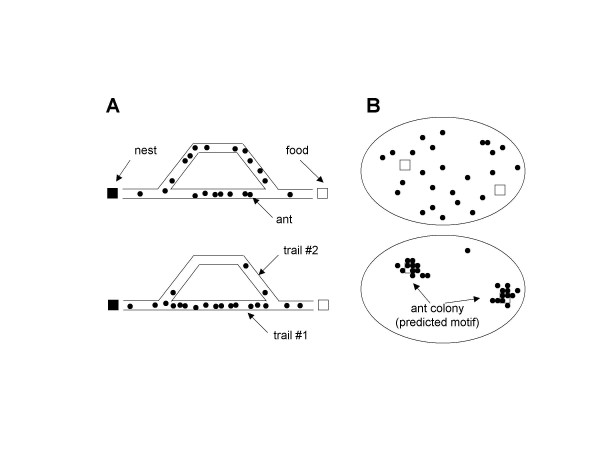
**Schematic illustration of the ant algorithm**. (A) Selection of a shorter path between a nest and a food source by natural ants. The ants travel between the nest and food through trail #1 and trail #2. Initially, ants are distributed equally on both trails (top). Since trail #1 is shorter than trail #2, trail #1 becomes their favorite pathway with a higher pheromone concentration (bottom). (B) Modeling gene expression using artificial ants. In an artificial nucleus consisting of two transcription-factor binding motifs involved in the regulatory model, ants are initially located in a random fashion (top). These two motifs are treated like a food source, and two ant colonies are formed after cycles of pheromone deposition and evaporation (bottom).

The ant algorithm is a branch of swarm intelligence techniques inspired by the foraging behavior of ant colonies. Here, the solution in a combinatorial problem is initialized with a population of randomly positioned ants. Each ant represents a particular solution and deposits a varying amount of artificial pheromone based on the fitness of the solution. The concentrations of pheromone provide distributed information in a whole solution space, and they are constantly updated through deposition and evaporation. With a positive feedback mechanism through reinforcement of the previously favored solutions, algorithm-based ants are guided towards the solution of higher fitness like the social behavior of natural ants (Fig. 1).

The ant algorithm is designed to predict a set of transcription-factor binding motifs using array-derived gene expression data. Based on the idea that gene expression levels are regulated by the combinatorial actions of multiple transcription-factor binding motifs, we formulated a linear relationship between the observed gene expression patterns and the appearance number of potential transcription-factor binding sites in the regulatory region of each gene. Thus, the ant algorithm seeks the motifs whose occurrences in the regulatory region collectively correlate with the global expression levels.

We examined whether the unique version of ant algorithms presented here would predict a potential contribution of all conceivable transcription-factor binding motifs. Most of the existing methods evaluate either a single oligo sequence at a time [11-14], or a fixed set of multiple binding motifs with little information on a role of individual motifs [14,15]. We previously utilized the genetic algorithm [15] as well as a particle swarm optimization procedure [14], and searched for a suboptimal combination of binding motifs. Such approaches, however, were not ideal because of the astronomical number of possibilities in the solution space (motif combinations) and the limited scope in the presence of redundant regulatory mechanisms. Here, we describe a novel application of ant algorithms to evaluate the role of each of individual binding motifs from a population of random DNA sequences. Unlike the genetic algorithm or particle swarm optimization, the ant algorithm aims to evaluate individual binding sites rather than searching for the best motif combination using a unique artificial pheromone spectrum.

In the current study prediction of transcription-factor binding motifs was formulated as a foraging problem of artificial ants using human chondrogenesis *in vitro *as a model system. Each ant represented a set of random DNA sequences and deposited a varying amount of artificial pheromones depending on the deviation from the array-derived data, which is defined in Eq. 4 later in the section of Methods. Pheromones were constantly renewed by deposition as well as partial evaporation, and ants were attracted to DNA sequences with high concentration of pheromones. This reinforcement process was aimed to select the most desirable group of transcription-factor binding motifs from random DNA sequences.

The microarray-derived mRNA expression data, being used throughout the study, were published by Sekiya *et al*. [16] (see additional file 1). The prediction of transcription-factor binding motifs was conducted previously with the genetic algorithm, and the results were experimentally validated using the genes specific to chondrogenesis such as type II collagen [15]. Here, we extended our analysis by developing a unique version of ant algorithms and evaluated the potential role of all DNA sequences in the solution space. All conceivable random sequences from 4 bp to 7 bp in length were analyzed, although the results with 5-bp DNA sequences were mainly described to validate the novel ant algorithm using the previous results with the genetic algorithm. The TRANSFAC database was used to derive biologically known consensus sequences (ranging from 5 to 30 bp) [17].

## Results

The overall strategy with the ant algorithm is to evaluate individual motif candidates considering combinatorial effects with other motifs. Briefly, each ant is defined to represent a potential combination of multiple transcription-factor binding motifs where the number of ants was determined based on the statistical studies with Akaike information criterion [15,18]). Using a mathematical model for expression profiles (Eqs. 1–3), each ant is associated with a model error (Eq. 4). A pheromone concentration is assigned high if the model error of the ant is small and vice versa (Eq. 5). In the next iteration of ant migration, the probability of particular motifs being selected by any ant is affected by the previous pheromone concentration (Eq. 6). The pheromone concentration deposited to each motif candidate is additive and evaporative (Eq. 7).

In the current study, 100 ants (*N*_*A *_= 100) were utilized with 1000 computational iterations for deposition and evaporation of pheromones. The final pheromone concentrations assigned to individual DNA sequences were plotted in a form of pheromone spectrum (See Methods). Two key parameters here were a "pheromone preference factor (*ε*)" and a "pheromone evaporation factor (*δ*)": the pheromone preference factor regulated affinity of ants towards artificial pheromone, and the pheromone evaporation factor was used to reduce influence from the earlier computational outcomes. Using these two factors, "reproducibility (*r*)" and "selectivity (*s*)" of the motif selections were analyzed. Reproducibility was defined as cross-correlation among the pheromone spectra, and selectivity was defined as "1 – information entropy" (See Methods).

### Pheromone spectrum

**Figure 2:**
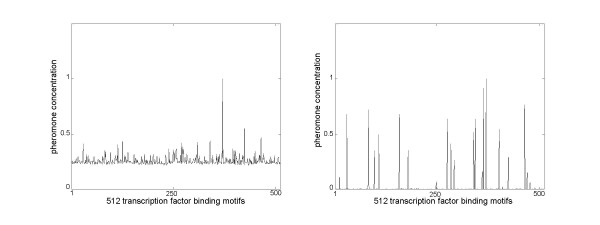
**Examples of pheromone spectra**. The pheromone spectra display the pheromone concentration for each DNA sequence as a potential transcription-factor binding motif. (A) Pheromone spectrum with *ε *= 0 and *δ *= 0. A single dominating peak indicates a highly selective computational condition. (B) Pheromone spectrum with *ε *= 500 and *δ *= 0.5. Multiple peaks suggest a distributed regulatory system with the given computational parameters.

Using 512 random DNA sequences of 5 bp in length as potential transcription-factor binding motifs, the fitness of individual motif candidates was analyzed from their final pheromone concentrations (Fig. 2). In the pheromone spectrum that illustrated 512 pheromone concentrations assigned to the sequences such as AAAAA, AAAAC, etc., they varied considerably depending on the choice of the pheromone preference factor (*ε*) and the pheromone evaporation factor (*δ*). With *ε *= 0 and *δ *= 0 (Fig. 2A), most DNA sequences received a relatively uniform pheromone concentration (0.25 ± 0.05; mean ± s.d.) except for CTGAC. Note that the highest concentration was normalized to 1. With *ε *= 500 and *δ *= 0.5 (Fig. 2B), on the other hand, 11 DNA fragments including CTGAC received the concentration higher than 4 s.d. above the average. These two examples indicated dependence of the pheromone spectrum on selection of *ε *and *δ *.

### Reproducibility and selectivity

**Figure 3:**
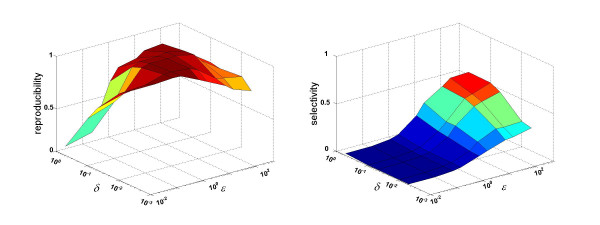
**Reproducibility and selectivity**. Reproducibility and selectivity of the described ant algorithm were characterized by defining two variables with 0 ≤ *ε *≤ 100 and 0 ≤ *δ *≤ 1. (A) Reproducibility defined as correlation between pheromone spectra in Eq. 8. (B) Selectivity defined as modified informational entropy in Eq. 9. The higher value represents a fewer peaks in the pheromone spectrum.

In order to characterize the effects of *ε *and *δ *in the ant algorithm, reproducibility (*r*) and selectivity (*s*) were determined for varying pairs of *ε *and *δ *for 0 ≤ *ε *≤ 100 and 0 ≤ *δ *≤ 1 (Fig. 3). As expected from random selection, the selectivity value became ~0 with *ε *~ 0 (no preference to pheromone). The selectivity value was high for a pair of large *ε *and small *δ*, while the reproducibility value approached 0.9 or above for 0.1 <*ε *< 10 and *δ *< 0.5. Based on statistical considerations, we aimed at high reproducibility and significantly low selectivity that would provide ~10 local peaks in the pheromone spectrum. Selecting of 10 local peaks corresponds to building a model with 10 potential binding motifs. Hereafter, a pair of *ε *= 10 and *δ *= 0.1 was employed to achieve *r *= 0.98 and *s *= 0.17 for modeling human chondrogenesis *in vitro*.

### Motif length analysis

**Figure 4:**
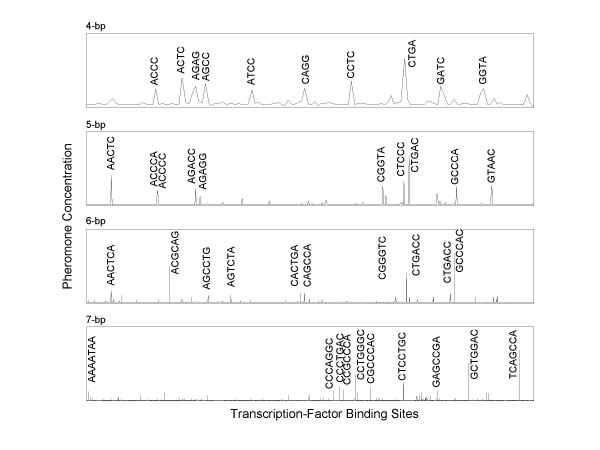
**Motif length analysis**. The motif length analysis among the four models (4-, 5-, 6-, and 7-bp DNA sequences as potential transcription-factor binding motifs) with *ε *= 10 and *δ *= 0.1. (A) Pheromone spectrum for 4-bp DNA sequences. (B) Pheromone spectrum for 5-bp DNA sequences. (C) Pheromone spectrum for 6-bp DNA sequences. (D) Pheromone spectrum for 7-bp DNA sequences.

Since the length of known transcription-factor binding motifs vary from 4 bp up to more than 10 bp, we examined sequence similarities among the predicted motifs ranging from 4 to 7 bp (Fig. 4). There are 136, 512, 2080, and 8192 DNA sequences in total for 4-, 5-, 6-, and 7-bp binding motifs, respectively. Interestingly, the motifs consisting of particular DNA sequences such as GCCC, CAGG, and CTGA repeatedly appeared with a high concentration of pheromone.

### Biological relevance

**Figure 5:**
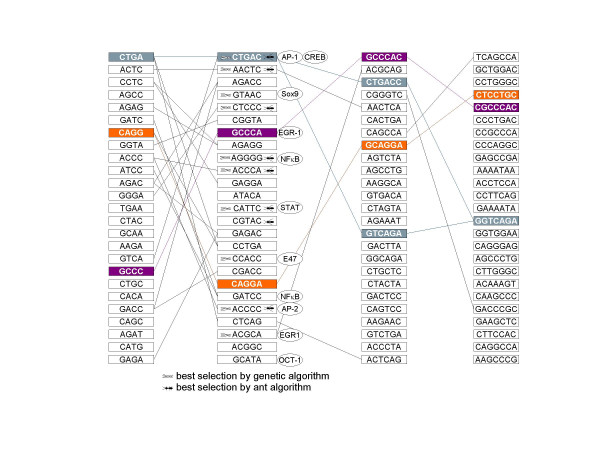
**Biological relevance**. Linkage of the transcription-factor binding motifs among the ant algorithm prediction, the genetic algorithm prediction, and the biologically known binding motifs.

Among the four models (4 to 7 bp motifs), the most influential 25 transcription-factor binding motifs were selected from a pool of 10,920 potential motifs (Fig. 5). First, 4-bp DNA sequences such as CTGA, CAGG, and GCCC were selected in all four models. To test statistical significance of selecting any 4-bp core sequence throughout the four models, Monte Carlo simulations were conducted. The simulation result supported statistical significance (*p *< 10^-5^) of picking three 4-bp core sequences from 4 to 7 bp motifs (25 motifs in each group), although a null hypothesis assumed here was significantly weak because of a multiple motif selection. Second, the binding motifs such as AP-1, Sox9 and NFκB known to be involved in chondrogenesis were selected in the 5-bp model (Table 1). Third, nine out of 10 binding motifs, predicted previously by the genetic algorithm, were included in this short list.

**Figure 6:**
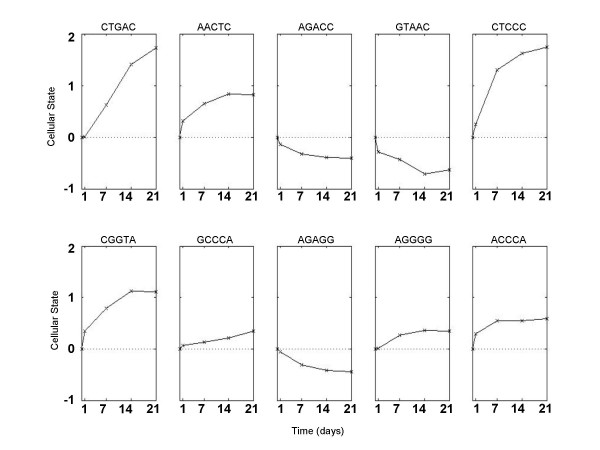
**State transition of the predicted binding motifs**. The predicted state levels are plotted on days 1, 7, 14, and 21 during *in vitro *chondrogenesis. The positive level indicates stimulation, and the negative level shows inhibition. The 5-bp DNA sequences, predicted with the ant algorithm, include CTGAC, AACTC, AGACC, GTAAC, CTCCC, CGGTA, GCCCA, AGAGG, AGGGG, and ACCCA.

The state transition representing the time-dependent role of the predicted transcription-factor binding motifs are illustrated on days 1, 7, 14, and 21 (Fig. 6). The figure reveals that DNA sequences such as CGTAC and AACTC were predicted to up-regulate the selected genes in the model, while the sequences such as ACCCA and AACAT were modeled to down-regulate the same genes. In particular, the predicted role of AGGGG is consistent to the molecular experiments using a transient DNA transfection [15].

## Discussion

This study described a novel application of the ant algorithm in predicting a role of each of the random DNA sequences as a transcription-factor binding motif in human chondrogenesis. The prediction procedure was formulated as the combinatorial problem to select a set of multiple motifs followed by a histogram analysis to build a spectrum of potential contributions among all conceivable motifs. Using human chondrogenesis *in vitro *as a model system, we demonstrate that the ant algorithm is capable of identifying DNA sequences found in the biologically known motifs such as AP-1, CREB, Sox9, NFκB, Erg-1, AP-2, Stat, Smad, E47, and Oct-1 as well as unknown candidates. We discuss the described ant algorithm focusing on its characteristic formulation, selectivity and reproducibility, computational efficiency, and biological relevance.

The first feature of the described algorithm is definition of artificial ants as a set of *m *transcription-factor binding motifs (*m *= 10 in this study). In this algorithm, each ant was assigned to its own set of *m *binding motifs and it deposited an equal amount of pheromone to the assigned set. A pheromone spectrum was then built from the sum of deposited pheromones by *N*_*A *_ants (*N*_*A *_= 100). The value of *m *> 1 allowed us not only to evaluate a combinatorial effect of multiple binding motifs but also to reduce the number of computational iterations.

The second feature among biology-inspired algorithms is a well-characterized choice of selectivity and reproducibility by the two key parameters: pheromone preference factor, *ε*, and pheromone evaporation factor, *δ*. The value of *ε *determines the preference to pheromones, and the value of *δ *regulates fading of the previous concentration of pheromone. Selectivity was defined as "1 – informational entropy" to evaluate distance from randomness, while reproducibility was defined as correlation among the spectra. A higher selectivity in general implies that the limited number of motif candidates receive a significantly higher pheromone concentration than most of the other candidates. The extreme case for *ε *~ 0 or *ε *> 100 yielded low reproducibility, since the final pheromone spectrum was predominantly influenced by the ants in the first or the last generation. A value of *δ *controlled evaporation of previous information, and a large value of *δ *placed more emphasis on recent decisions. The values of *ε *and *δ *can be selected arbitrarily depending on the purpose of a particular study. In this study we determined these parameters to reproducibly obtain ~10 peaks in the spectrum. The number of peaks was pre-determined using Akaike information criterion [18].

A clear advantage of the ant algorithm is a computational efficiency to reach a stable solution compared to the other evolutionary algorithms such as the genetic algorithm. The major difference between the ant algorithm and the genetic algorithm is their solution space. The ant algorithm searches for a group of the best motifs in a space of individual binding motifs, while the genetic algorithm seeks the best combination. The former space is apparently more restricted than the latter combinatorial space. Therefore, the ant algorithm has a clear advantage to terminate the search. Furthermore, the advantage of the ant algorithm includes identification of redundant transcription-factor binding motifs in eukaryotic gene regulation. Neither the genetic algorithm nor particle swarm optimization is well suited to include redundant motifs in a final solution.

The ant algorithm is also different from other model-based approaches such as REDUCE [11] and the principal component analysis [14]. In REDUCE, for instance, motifs are selected recursively in an add-on manner to reduce the model error by the largest degree at each selection. This selection strategy makes later selections strongly affected by the earlier ones. The ant algorithm, on the other hand, can avoid such a potential conflict in selection. In order to compare these two algorithms, numerical simulations were conducted using a benchmark dataset for 200 artificial genes (see additional file 1). Simulation results suggested that both REDUCE and ant algorithm offers similar power in predicting correct number of embedded motifs. Although the advantage of REDUCE is its superior reproducibility with a shorter computational time, the ant algorithm is apparently more suited to predict a motif longer than 5 bp including a motif consisting of a dimeric binding site. Biologists are usually advised to use several complementary computational tools to identify regulatory elements from microarray data [19]. The ant algorithm seems to complement analytical approaches such as REDUCE and the principal component-based method in identifying longer motifs and dimeric binding sites.

The ant algorithm can still evaluate combinatorial effects among multiple factors like other model-based approaches reported previously [11,14-16]. Defining a background model is a general approach in searching for over-representation of DNA words within a sequence set. The ant algorithm, however, aims at searching for the DNA sequences whose occurrences in the regulatory region correlate with the observed expression levels in the context of combinations of multiple motifs. Therefore, the described ant algorithm is in principle not overly sensitive to over-represented DNA words. The expression levels are described as a linear combination of the role of individual motifs with different functions. Therefore, it is possible that some motifs, predicted to be a stimulator by the ant algorithm, may appear in the regulatory region of the gene whose expression level is down-regulated.

A motif length analysis and sequence comparisons supported, at least in part, statistical and biological significance of the selected transcription-factor binding motifs. The 25 predicted motifs matched with the sequences of 10 known binding motifs known to be involved in human chondrogenesis. These binding motifs include AP-1, CREB, Sox9, NFκB, Erg-1, AP-2, Stat, Smad, E47, and Oct-1. Interestingly, two of the 5-bp motifs (GCCCA and ACGCA) together with a 6-bp motif (GCCCAC) and a 7-bp motif (CGCCCAC) constituted a contiguous 10-bp binding motif of Egr-1 ([A/T]TGCGTGGGCG [G/T]), confirming a strong involvement of Egr-1 in chondrogenesis. Furthermore, two 5-bp motifs (GATCC and AGGGG) coincided with 9-bp consensus sequence of NFκB (p50) binding sites (GGGGAT [C/T]CCCC [A/T]NTC [C/G]). It is possible to evaluate a pool of candidates with varying length simultaneously by including them together in the simulation. Note that the prediction by the ant algorithm should be used to address a set of hypotheses, and biological experiment is inevitable.

In summary, the described procedure is the first application of ant algorithms for prediction of transcription-factor binding motifs. Other definitions of artificial ants and pheromones are possible. For instance, a group of heterogeneous ants could behave like transcription factors or RNA polymerases and deposit different kinds of pheromones directly onto genomic DNA sequences. We believe that this application will be advanced by further studies for improving computational efficiency and biological relevance.

## Conclusion

We developed one form of ant algorithms for prediction of transcription-factor binding motifs. The consensus sequences of 10 biologically known binding motifs have significant similarities with the predicted motifs. Unlike healing capability of bones, joint tissues such as articular cartilage hardly regenerate and therefore *in vitro *chondrogenesis is an extremely challenging subject in tissue engineering. The transcriptional mechanism of human chondrogenesis remains largely unknown. With its efficient search procedure and its controllable reproducibility and selectivity, the described version of ant algorithms allows us to provide a known and novel set of molecular targets for biological verification.

## Methods

### Biological model system

We focused our analysis on 55 genes whose alterations in mRNA were statistically significant during human chondrogenesis as published by Sekiya *et al*. in Table 1[16], and their 5'-end flanking DNA sequences were identifiable with the UCSC genome browser (see additional file 1). The logarithmic ratios in the gene expression levels on days 1, 7, 14, and 21 relative to day 0 were used in the model:

zi(t)=log2yi(t)yi(0)     (1)
 MathType@MTEF@5@5@+=feaafiart1ev1aaatCvAUfKttLearuWrP9MDH5MBPbIqV92AaeXatLxBI9gBaebbnrfifHhDYfgasaacH8akY=wiFfYdH8Gipec8Eeeu0xXdbba9frFj0=OqFfea0dXdd9vqai=hGuQ8kuc9pgc9s8qqaq=dirpe0xb9q8qiLsFr0=vr0=vr0dc8meaabaqaciaacaGaaeqabaqabeGadaaakeaacqWG6bGEdaWgaaWcbaGaemyAaKgabeaakiabcIcaOiabdsha0jabcMcaPiabg2da9Gqaciab=XgaSjab=9gaVjab=DgaNnaaBaaaleaacqaIYaGmaeqaaOWaaSaaaeaacqWG5bqEdaWgaaWcbaGaemyAaKgabeaakiabcIcaOiabdsha0jabcMcaPaqaaiabdMha5naaBaaaleaacqWGPbqAaeqaaOGaeiikaGIaeGimaaJaeiykaKcaaiaaxMaacaWLjaWaaeWaceaacqaIXaqmaiaawIcacaGLPaaaaaa@48D3@

where *y*_*i*_(*t*) = mean mRNA level of the i-th gene on day 1, 7, 14 or 21, and = *y*_*i*_(0) mean mRNA level of the i-th gene on day 0. The positive and negative ratios indicate upregulation and downregulation to day 0, respectively.

### Model-based analysis

The global gene expression patterns were modeled using the number of occurrences of the potential transcription-factor binding sites in the 5'-flanking regulatory region of the gene:

*Z*_*n*_(*t*) = *H*_*nxm *_*X*_*m*_(*t*)     (2)

where *Z*_*n*_(*t*) represents logarithmic ratios of differential gene expression levels derived in Eq. 1, the element *h*_*ij *_in *H*_*nxm *_denotes the number of j-th transcription-factor binding motif in the regulatory region of the *i*-th gene, and *X*_*m*_(*t*) corresponds to the functional levels of *m *predicted binding sites at time *t*. The positive and negative values in *X*_*m*_(*t*) suggest stimulatory and inhibitory roles of the corresponding transcription-factor binding motif, respectively. Here, *n *is the number of genes. The upstream regulatory sequence of each gene was acquired from the USCS genome browser. Based on the results of our previous studies [15,16] and others [20,21], we used the 1000-bp upstream region of the transcription starting site (see additional file 1). This region, however, should be considered as a parameter to be chosen, and the model can include further upstream regions, downstream regions, or untranslated regions. As potential transcription-factor binding motifs, a complete set of random DNA sequences of 4 to 7 bp in length (AAAA, AAAC, AAAG, AAAT, etc.) was considered and their distribution was identified on the 5'-end flanking region of the genes in the model. In formulation of Eq. 2, the reverse complementary motifs were combined with their counterparts. Namely, we counted the appearances of both the forward and the reverse motifs and treated them together as a single motif candidate. Therefore, the total numbers of 4-, 5-, 6-, and 7-bp motifs are 136, 512, 2080, and 4192, respectively.

We evaluated a set of random sequences in two steps. First, the functional level of each motif (*x *value) was estimated using a least-square procedure (Eq. 3). Second, the cost function of each set was defined as sum square error of the differences between the experimental and the predicted gene expression levels (Eq. 4):

X^m(t)=(HnxmTHnxm)−1HnxmTZn(t)     (3)
 MathType@MTEF@5@5@+=feaafiart1ev1aaatCvAUfKttLearuWrP9MDH5MBPbIqV92AaeXatLxBI9gBaebbnrfifHhDYfgasaacH8akY=wiFfYdH8Gipec8Eeeu0xXdbba9frFj0=OqFfea0dXdd9vqai=hGuQ8kuc9pgc9s8qqaq=dirpe0xb9q8qiLsFr0=vr0=vr0dc8meaabaqaciaacaGaaeqabaqabeGadaaakeaacuWGybawgaqcamaaBaaaleaacqWGTbqBaeqaaOGaeiikaGIaemiDaqNaeiykaKIaeyypa0JaeiikaGIaemisaG0aa0baaSqaaiabd6gaUjabdIha4jabd2gaTbqaaiabdsfaubaakiabdIeainaaBaaaleaacqWGUbGBcqWG4baEcqWGTbqBaeqaaOGaeiykaKYaaWbaaSqabeaacqGHsislcqaIXaqmaaGccqWGibasdaqhaaWcbaGaemOBa4MaemiEaGNaemyBa0gabaGaemivaqfaaOGaemOwaO1aaSbaaSqaaiabd6gaUbqabaGccqGGOaakcqWG0baDcqGGPaqkcaWLjaGaaCzcamaabmGabaGaeG4mamdacaGLOaGaayzkaaaaaa@544F@

ek=∑i=1n∑t=1,7,14,21[zi(t)−z^i(t)]2     (4)
 MathType@MTEF@5@5@+=feaafiart1ev1aaatCvAUfKttLearuWrP9MDH5MBPbIqV92AaeXatLxBI9gBaebbnrfifHhDYfgasaacH8akY=wiFfYdH8Gipec8Eeeu0xXdbba9frFj0=OqFfea0dXdd9vqai=hGuQ8kuc9pgc9s8qqaq=dirpe0xb9q8qiLsFr0=vr0=vr0dc8meaabaqaciaacaGaaeqabaqabeGadaaakeaacqWGLbqzdaWgaaWcbaGaem4AaSgabeaakiabg2da9maaqahabiqaaScddaaeqbqaamaadmGabaGaemOEaO3aaSbaaSqaaiabdMgaPbqabaGccqGGOaakcqWG0baDcqGGPaqkcqGHsislcuWG6bGEgaqcamaaBaaaleaacqWGPbqAaeqaaOGaeiikaGIaemiDaqNaeiykaKcacaGLBbGaayzxaaWaaWbaaSqabeaacqaIYaGmaaaabaGaemiDaqNaeyypa0JaeGymaeJaeiilaWIaeG4naCJaeiilaWIaeGymaeJaeGinaqJaeiilaWIaeGOmaiJaeGymaedabeqdcqGHris5aaWcbaGaemyAaKMaeyypa0JaeGymaedabaGaemOBa4ganiabggHiLdGccaWLjaGaaCzcamaabmGabaGaeGinaqdacaGLOaGaayzkaaaaaa@58F0@

where *e*_*k *_is the cost function of the *k*-th ant, *z*_*i*_(*t*) and z^
 MathType@MTEF@5@5@+=feaafiart1ev1aaatCvAUfKttLearuWrP9MDH5MBPbIqV92AaeXatLxBI9gBaebbnrfifHhDYfgasaacH8akY=wiFfYdH8Gipec8Eeeu0xXdbba9frFj0=OqFfea0dXdd9vqai=hGuQ8kuc9pgc9s8qqaq=dirpe0xb9q8qiLsFr0=vr0=vr0dc8meaabaqaciaacaGaaeqabaqabeGadaaakeaacuWG6bGEgaqcaaaa@2E39@_*i*_(*t*) are the observed and the predicted gene expression vectors in *Z*_*n*_(*t*) and Z^
 MathType@MTEF@5@5@+=feaafiart1ev1aaatCvAUfKttLearuWrP9MDH5MBPbIqV92AaeXatLxBI9gBaebbnrfifHhDYfgasaacH8akY=wiFfYdH8Gipec8Eeeu0xXdbba9frFj0=OqFfea0dXdd9vqai=hGuQ8kuc9pgc9s8qqaq=dirpe0xb9q8qiLsFr0=vr0=vr0dc8meaabaqaciaacaGaaeqabaqabeGadaaakeaacuWGAbGwgaqcaaaa@2DF9@_*n*_(*t*), respectively.

### Ant algorithm

From the group of random DNA sequences, the ant algorithm with *N*_*A *_ants was used to select the set of *m *transcription-factor binding motifs that would minimize the cost function. The ant algorithm in this study included three steps such as deposition of pheromones, pheromone-guided selection, and evaporation of pheromones.

Deposition of pheromones – First, each ant was assigned *m *random DNA sequences as potential transcription-factor binding motifs and evaluated from its cost function in Eq. 4 [22,23]. Based on their cost performance in Eq. 4, *N*_*A *_ants deposited the same amount of pheromone to each assigned DNA sequence. At the i-th iteration, for instance, the amount of deposition on each motif was defined:

fj,i=∑k∈Kj,i1ekα     (5)
 MathType@MTEF@5@5@+=feaafiart1ev1aaatCvAUfKttLearuWrP9MDH5MBPbIqV92AaeXatLxBI9gBaebbnrfifHhDYfgasaacH8akY=wiFfYdH8Gipec8Eeeu0xXdbba9frFj0=OqFfea0dXdd9vqai=hGuQ8kuc9pgc9s8qqaq=dirpe0xb9q8qiLsFr0=vr0=vr0dc8meaabaqaciaacaGaaeqabaqabeGadaaakeaacqWGMbGzdaWgaaWcbaGaemOAaOMaeiilaWIaemyAaKgabeaakiabg2da9maaqafabaWaaSaaaeaacqaIXaqmaeaacqWGLbqzdaqhaaWcbaGaem4AaSgabaacciGae8xSdegaaaaaaeaacqWGRbWAcqGHiiIZcqWGlbWsdaWgaaadbaGaemOAaOMaeiilaWIaemyAaKgabeaaaSqab0GaeyyeIuoakiaaxMaacaWLjaWaaeWaceaacqaI1aqnaiaawIcacaGLPaaaaaa@4615@

where *f*_*j*,*i *_= amount of pheromone deposited to the j-th potential binding motif by the i-th ant, *K*_*j*,*i *_= all the ants that host the j-th motif in the i-th iteration, *e*_*k *_= cost function of the k-th ant derived from Eq. 4, and *α *= power factor for error evaluation (*α *> 1).

Pheromone-guided selection – Based on the pheromone concentration assigned to each of the potential transcription-factor binding motifs, each of *N*_*A *_ants selected *m *DNA sequences at the (i+1)-th iteration:

pj,i+1=1M+εFj,i∑j=1MFj,i1+ε     (6)
 MathType@MTEF@5@5@+=feaafiart1ev1aaatCvAUfKttLearuWrP9MDH5MBPbIqV92AaeXatLxBI9gBaebbnrfifHhDYfgasaacH8akY=wiFfYdH8Gipec8Eeeu0xXdbba9frFj0=OqFfea0dXdd9vqai=hGuQ8kuc9pgc9s8qqaq=dirpe0xb9q8qiLsFr0=vr0=vr0dc8meaabaqaciaacaGaaeqabaqabeGadaaakeaacqWGWbaCdaWgaaWcbaGaemOAaOMaeiilaWIaemyAaKMaey4kaSIaeGymaedabeaakiabg2da9maalaaabaWaaSaaaeaacqaIXaqmaeaacqWGnbqtaaGaey4kaSccciGae8xTdu2aaSaaaeaacqWGgbGrdaWgaaWcbaGaemOAaOMaeiilaWIaemyAaKgabeaaaOqaamaaqahabaGaemOray0aaSbaaSqaaiabdQgaQjabcYcaSiabdMgaPbqabaaabaGaemOAaOMaeyypa0JaeGymaedabaGaemyta0eaniabggHiLdaaaaGcbaGaeGymaeJaey4kaSIae8xTdugaaiaaxMaacaWLjaWaaeWaceaacqaI2aGnaiaawIcacaGLPaaaaaa@513E@

where *p*_*j*,*i*+1 _= probability of selecting the j-th binding motif, *ε *= pheromone preference factor (*ε *>0), and *F*_*j*,*i *_= cumulative pheromone concentration of the j-th binding motif. Note that *M *is the total number of potential transcription-factor binding motifs, and it is 136, 512, 2080, and 8192 for 4-, 5-, 6-, and 7-bp selections, respectively. When *ε *= 0, the selection of DNA sequences would be conducted randomly without any preference to pheromones.

Evaporation of pheromones – The pheromone concentration, *F*_*j*,*i*_, was updated at each iteration step:

*F*_*j*,*i*+1 _= (1 - *δ*)*F*_*j*,*i *_+*f*_*j*,*i *_    (7)

where *δ *= pheromone evaporation factor (0 ≤ *δ *≤ 1). When *δ *= 0, the pheromone would be preserved without evaporation. On contrary, the previous pheromone information was completely lost with *δ *= 1.

### Evaluation of reproducibility and selectivity

Two key parameters in the ant algorithm were *ε *(pheromone preference factor), and *δ *(pheromone evaporation factor). In order to evaluate the role of these two parameters in reproducibility and selectivity of transcription-factor binding motifs, we defined reproducibility, *r *(0 ≤ *r *≤ 1), and selectivity, *s *(0 ≤ *s *≤ 1):

*r *= *ρ*(Φ, Φ')     (8)

s=1+1ln(M)∑j=1Mϕjln(ϕj)     (9)
 MathType@MTEF@5@5@+=feaafiart1ev1aaatCvAUfKttLearuWrP9MDH5MBPbIqV92AaeXatLxBI9gBaebbnrfifHhDYfgasaacH8akY=wiFfYdH8Gipec8Eeeu0xXdbba9frFj0=OqFfea0dXdd9vqai=hGuQ8kuc9pgc9s8qqaq=dirpe0xb9q8qiLsFr0=vr0=vr0dc8meaabaqaciaacaGaaeqabaqabeGadaaakeaacqWGZbWCcqGH9aqpcqaIXaqmcqGHRaWkdaWcaaqaaiabigdaXaqaaGqaciab=XgaSjab=5gaUjabcIcaOiabd2eanjabcMcaPaaadaaeWbqaaGGaciab+v9aQnaaBaaaleaacqWGQbGAaeqaaaqaaiabdQgaQjabg2da9iabigdaXaqaaiabd2eanbqdcqGHris5aOGae8hBaWMae8NBa4MaeiikaGIae4x1dO2aaSbaaSqaaiabdQgaQbqabaGccqGGPaqkcaWLjaGaaCzcamaabmGabaGaeGyoaKdacaGLOaGaayzkaaaaaa@4D3B@

where *ρ *= correlation coefficient between two pheromone spectra Φ and Φ', *M *= total number of DNA sequences in the model, and *φ*_*j *_= final pheromone concentration of the *j*-th potential transcription-factor binding motif. With *r *= 1, two pheromone spectra become identical. The selectivity parameter was defined as "1 – informational entropy," and with *s *= 1 only one binding motif received pheromones with no pheromone deposition on others. Note that the similar definition of informational entropy was first employed to evaluate variations in expression profiles [22].

### Monte Carlo simulation and comparison to TRANSFAC database

The independent models using a different length of DNA sequences as potential transcription-factor binding motifs resulted in several common core DNA sequences. In order to evaluate statistical significance of identifying 4-bp core sequences, Monte Carlo simulation was conducted. First, 25 DNA sequences were randomly selected in each of the models with 4-, 5-, 6-, or 7-bp binding motifs. Then, the number of 4-bp common DNA sequences in the four models was counted. This procedure was repeated for 1,000 times, and a p-value for finding a particular number of 4-bp core sequences was evaluated. The predicted motifs were compared with the biologically known motifs in the TRANSFAC database using the procedure previously published [23].

### Comparison between the ant algorithm and REDUCE

In order to compare capabilities of the ant algorithm with REDUCE [11], a benchmark dataset consisting of 200 artificial genes was generated and numerical simulations were conducted. In the dataset the promoter sequences of 1000 bp in length were randomly generated and a set of hypothetical binding motifs (6–10 bp long) with a known functional level were embedded arbitrarily in the promoter sequences The expression levels of 200 genes were modelled using Eq. 2, and the predicted expression levels using the ant algorithm and REDUCE were evaluated (see additional file 1).

## Authors' contributions

Both authors (YL and HY) participated in the design of the study, analysis of the results and writing of the manuscript. Both authors read and approved the final manuscript.

## Supplementary Material

Additional File 1 • Part I – Comparison between the ant algorithm and REDUCE. • Part II – List of genes included in the model. • Part III – Regulatory DNA sequences (1000 bp upstream of the transcription starting site) used in the model. Click here for file

## Figures and Tables

**Table 1:** Comparison of the predicted binding motifs to the biologically known motifs

Known binding sites	Consensus sequences	Predicted motifs
AP-1	TGA**GTCAK**CNTGA	CTGAC
CREB	C**GTCAN**RGGC	CTGAC
SOX9	G**GTAAC**AAGA	GTAAC
EGR-1	W**TGCGTGGGC**GK	GCCCA, ACGCA
AP-2	G**GSGGT**GMNTTCC	ACCCC
STAT	TTCCCG**KAATG**GM	CATTC
SMAD3	T**GTCTG**TCTCTGACSTCA	GAGAC
E47	VSNGCA**GGTGK**	CCACC
OCT1	**TATGC**AAAT	GCATA
NFκB	GGG**GATYCCCCW**N	GATCC, AGGGG
