# Supplementary material for: Artificial ants deposit pheromone to search for regulatory DNA elements
Source: BMC Genomics. 2006 Aug 30;7:221. doi: 10.1186/1471-2164-7-221 (PMC1586019; doi:10.1186/1471-2164-7-221)
Supplement: Additional File 1 — • Part I – Comparison between the ant algorithm and REDUCE. • Part II – List of genes included in the model. • Part III – Regulatory DNA sequences (1000 bp upstream of the transcription starting site) used in the model. [file 1471-2164-7-221-S1.doc]

**Additional File**

This supplementary information includes the following three parts:

- Part I Comparison between the ant algorithm and REDUCE
- Part II List of the genes included in the model
- Part III Regulatory DNA sequences (1000 bp upstream sequences from the

transcription starting site) used in the model

**Part I – Comparison between the ant algorithm and REDUCE**

In the first part, we constructed a benchmark dataset to compare the predictions from the ant algorithm and REDUCE [1].

Methods

1. Generate a set of random promoters.

We randomly generated a set of 200 promoter sequences of 1000 nucleotides long, using appropriate AT and GC contents observed in human genome.

1. Embed multiple artificial motifs.

Ten artificial motifs (6 to 12 bp in length) as well as 4 heterodimeric motifs (6 bp in monomeric length each) were selected with their functional level, and they were randomly embedded into the promoter sequences.

1. Calculate an expression profile for each promoter.

The expression level of each promoter was calculated based on the number of artificial motifs and their assigned functional level (Eq. 2).

1. Predict binding motifs using the ant algorithm and REDUCE.

The ant algorithm and REDUCE were applied to predict motif candidates for the above expression system. The 5-bp motifs (top 20 candidates) selected by each of the approach were compared with the embedded motifs.

Results

The simulation was conducted 100 times. Generally, the numbers of correct predictions from the ant algorithm and REDUCE are not distinguishable. In 78 runs among the 100 simulations, the number of correct predictions of the two methods differed by no more than 2. Although these two algorithms identified a different set of motifs, no clear advantage of one approach to the other was observed in many cases.

The ant algorithm, however, showed its tendency to predict motifs longer than 5 bp. First, it can predict a longer motif by overlapping multiple 5-bp motifs. Second, it can predict a complete motif consisting of demeric binding sites. REDUCE, on the other hand, failed to encompass either the longer motif or the dimeric binding sites. One simulational example is shown in Figure S1:

(a) Among the 14 embedded motifs, REDUCE identified 4 motifs correctly and 1 motif partially. The ant algorithm predicted 6 motifs correctly. Among those 6 motifs, 2 long motifs were identified using two 5-bp candidates;

(b) The ant algorithm identified two subregions in the long motifs such as TTACGTAA and CCAATAATCGAT as well as two parts of a dimeric motif of TCGTAA (n)_1-5_ CCGTTA.

**
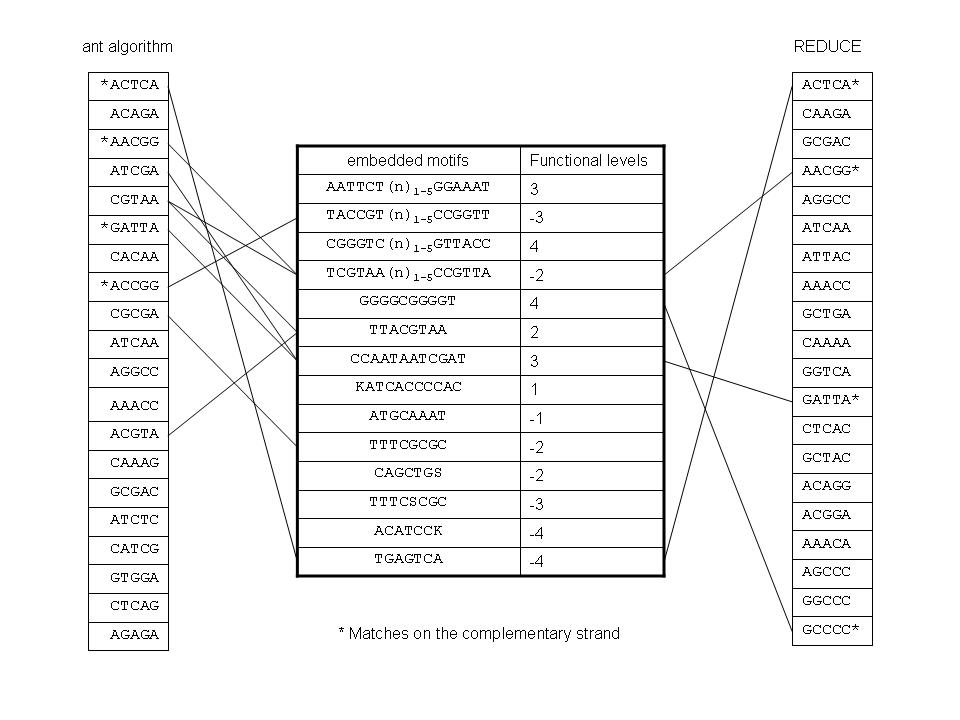
Figure S1. Comparison between ant algorithm and REDUCE**

Reference

1. Bussemaker HJ, Li H, Siggia ED**: Regulatory element detection using correlation with expressi**on*. Nat Gene*t 2001**,** 27(2):167-171.

**Part II – List of the genes that are included in the model**

| **GenBank** | **Gene** | **Fold changes vs. day 0** | | | |
| --- | --- | --- | --- | --- | --- |
|  |  | **Day 1** | **Day 7** | **Day 14** | **Day 21** |
| [M27492](http://www.pnas.org/cgi/external_ref?access_num=M27492&link_type=GEN) | IL1R1 | 7.7 | 8.6 | 9.3 | 8.4 |
| [AJ001047](http://www.pnas.org/cgi/external_ref?access_num=AJ001047&link_type=GEN) | Matrilin-3 | 1 | 4.8 | 34.3 | 140.9 |
| [U59289](http://www.pnas.org/cgi/external_ref?access_num=U59289&link_type=GEN) | Cadherin 13 | 1.1 | -1.5 | -6.3 | -7.6 |
| [X00129](http://www.pnas.org/cgi/external_ref?access_num=X00129&link_type=GEN) | RBP | 1 | 5.1 | 23.1 | 44.6 |
| [AB000114](http://www.pnas.org/cgi/external_ref?access_num=AB000114&link_type=GEN) | Osteomodulin | 1 | 12.5 | 38.4 | 58.6 |
| [L10347](http://www.pnas.org/cgi/external_ref?access_num=L10347&link_type=GEN) | COL2A1 | 1 | 49 | 358.8 | 530.7 |
| [X14420](http://www.pnas.org/cgi/external_ref?access_num=X14420&link_type=GEN) | COL3A1 | 2 | 11 | 12.1 | 9.2 |
| [X54412](http://www.pnas.org/cgi/external_ref?access_num=X54412&link_type=GEN) | COL9A1 | 2.6 | 2.4 | 6.1 | 12.7 |
| [M95610](http://www.pnas.org/cgi/external_ref?access_num=M95610&link_type=GEN) | COL9A2 | 1 | 27.6 | 63.4 | 68 |
| [L41162](http://www.pnas.org/cgi/external_ref?access_num=L41162&link_type=GEN) | COL9A3 | 1 | 25.5 | 104.4 | 172.7 |
| [J04177](http://www.pnas.org/cgi/external_ref?access_num=J04177&link_type=GEN) | COL11A1 | 5.8 | 122 | 217 | 243.5 |
| [M92642](http://www.pnas.org/cgi/external_ref?access_num=M92642&link_type=GEN) | COL16A1 | 1.1 | 4.1 | 5.6 | 4 |
| [L32137](http://www.pnas.org/cgi/external_ref?access_num=L32137&link_type=GEN) | COMP | 2 | 103.7 | 233.9 | 234.9 |
| [U05291](http://www.pnas.org/cgi/external_ref?access_num=U05291&link_type=GEN) | Fibromodulin | 1 | 7.6 | 47.6 | 100 |
| [J05213](http://www.pnas.org/cgi/external_ref?access_num=J05213&link_type=GEN) | Sialoprotein precursor | 1 | 1 | 3.8 | 45.8 |
| [AB011792](http://www.pnas.org/cgi/external_ref?access_num=AB011792&link_type=GEN) | ECM2 | 1.2 | 18.6 | 36.1 | 23.6 |
| [M55172](http://www.pnas.org/cgi/external_ref?access_num=M55172&link_type=GEN) | Aggrecan core protein | -2.1 | 8.7 | 17.2 | 19.1 |
| [AB006000](http://www.pnas.org/cgi/external_ref?access_num=AB006000&link_type=GEN) | Chondromodulin I precursor | 1 | 1 | 3.3 | 16.9 |
| [U69263](http://www.pnas.org/cgi/external_ref?access_num=U69263&link_type=GEN) | Matrilin-2 precursor | 1 | 3.1 | 3.8 | 5.4 |
| [Z46629](http://www.pnas.org/cgi/external_ref?access_num=Z46629&link_type=GEN) | SOX9 | 2.9 | 11.9 | 9.9 | 14.3 |
| [M97676](http://www.pnas.org/cgi/external_ref?access_num=M97676&link_type=GEN) | HOXA7 | -1.7 | 2.7 | 5.1 | 3.1 |
| [U43148](http://www.pnas.org/cgi/external_ref?access_num=U43148&link_type=GEN) | Patched | 1.9 | 1.5 | 2.6 | 6.4 |
| [Y12692](http://www.pnas.org/cgi/external_ref?access_num=Y12692&link_type=GEN) | WNT11 | 1 | 1.3 | 4.4 | 8.7 |
| [L37882](http://www.pnas.org/cgi/external_ref?access_num=L37882&link_type=GEN) | Frizzled-2 | -2.5 | -3.1 | -7.3 | -16.0 |
| [AF032885](http://www.pnas.org/cgi/external_ref?access_num=AF032885&link_type=GEN) | Forkhead | 25.4 | 76.1 | 58.5 | 62.6 |
| [Z50781](http://www.pnas.org/cgi/external_ref?access_num=Z50781&link_type=GEN) | hDIP | 18.6 | 16.3 | 13.1 | 29.2 |
| [AF009801](http://www.pnas.org/cgi/external_ref?access_num=AF009801&link_type=GEN) | BAPX1 | 1.4 | 1 | 2 | 5 |
| [J03779](http://www.pnas.org/cgi/external_ref?access_num=J03779&link_type=GEN) | CD10 | 3.8 | 7.6 | 2.8 | 2 |
| [AF039917](http://www.pnas.org/cgi/external_ref?access_num=AF039917&link_type=GEN) | CD39L3 | 1 | 1 | 2.5 | 7.2 |
| [L05424](http://www.pnas.org/cgi/external_ref?access_num=L05424&link_type=GEN) | CD44 | -1.6 | -2.7 | -7.1 | -5.7 |
| [M59911](http://www.pnas.org/cgi/external_ref?access_num=M59911&link_type=GEN) | Integrin alpha 3 | -1.8 | -7.4 | -10.4 | -6.6 |
| [M22489](http://www.pnas.org/cgi/external_ref?access_num=M22489&link_type=GEN) | BMP2 | 4.3 | 11.6 | 5.6 | 5.4 |
| [AF024710](http://www.pnas.org/cgi/external_ref?access_num=AF024710&link_type=GEN) | VEGF | 8.3 | 3.2 | 2.3 | 3.7 |
| [X94216](http://www.pnas.org/cgi/external_ref?access_num=X94216&link_type=GEN) | VEGF-C | -3.2 | -5.6 | -9.7 | -11.2 |
| [X00588](http://www.pnas.org/cgi/external_ref?access_num=X00588&link_type=GEN) | EGFR-precursor | 1.2 | -2.0 | -6.4 | -10.2 |
| [M37825](http://www.pnas.org/cgi/external_ref?access_num=M37825&link_type=GEN) | FGF5 | -11.5 | -11.5 | -11.5 | -11.5 |
| [Z71929](http://www.pnas.org/cgi/external_ref?access_num=Z71929&link_type=GEN) | FGFR2 | 1.1 | 3 | 14.7 | 14.9 |
| [X75308](http://www.pnas.org/cgi/external_ref?access_num=X75308&link_type=GEN) | MMP13 | 1 | 6.3 | 1.6 | 1.1 |
| [X83535](http://www.pnas.org/cgi/external_ref?access_num=X83535&link_type=GEN) | MMP14 | 6.2 | 2.2 | 1.7 | 1.3 |
| [D83646](http://www.pnas.org/cgi/external_ref?access_num=D83646&link_type=GEN) | MMP16 | 1 | 4.6 | 6.4 | 9.3 |
| [X17406](http://www.pnas.org/cgi/external_ref?access_num=X17406&link_type=GEN) | Aggrecan | 1 | 30.3 | 89.8 | 115.8 |
| [U90441](http://www.pnas.org/cgi/external_ref?access_num=U90441&link_type=GEN) | Prolyl 4-hydroxylase alpha 2 | 2.8 | 3.7 | 5.9 | 8.1 |
| [X70683](http://www.pnas.org/cgi/external_ref?access_num=X70683&link_type=GEN) | SOX4 | 7.9 | 5.3 | 3.3 | 1.7 |
| [S83308](http://www.pnas.org/cgi/external_ref?access_num=S83308&link_type=GEN) | SOX5 | 1 | 1.7 | 3.4 | 5 |
| [U17418](http://www.pnas.org/cgi/external_ref?access_num=U17418&link_type=GEN) | PTHrP-R | 1.5 | 21.2 | 70.4 | 127.1 |
| [L13463](http://www.pnas.org/cgi/external_ref?access_num=L13463&link_type=GEN) | G0S8 | 3.9 | 14.1 | 10.3 | 13.5 |
| [L33930](http://www.pnas.org/cgi/external_ref?access_num=L33930&link_type=GEN) | CD24 | 2.4 | 17.5 | 27.8 | 22.8 |
| [X57025](http://www.pnas.org/cgi/external_ref?access_num=X57025&link_type=GEN) | IGF1 | 1 | 2.9 | 5.4 | 4.2 |
| [M65062](http://www.pnas.org/cgi/external_ref?access_num=M65062&link_type=GEN) | IGFBP5 | 10.6 | 19.7 | 29.8 | 26.1 |
| [M62402](http://www.pnas.org/cgi/external_ref?access_num=M62402&link_type=GEN) | IGFBP6 | 1.6 | -1.4 | -4.2 | -6.1 |
| [L38517](http://www.pnas.org/cgi/external_ref?access_num=L38517&link_type=GEN) | IHH | 2.9 | 1 | 1 | 5.9 |
| [X14149](http://www.pnas.org/cgi/external_ref?access_num=X14885&link_type=GEN) | TGF-3 | 5 | 5.4 | 5.2 | 3.4 |
| [AB004922](http://www.pnas.org/cgi/external_ref?access_num=AB004922&link_type=GEN) | SMAD3 | -2.8 | -5.5 | -5.5 | -5.5 |
| [NM_001937](http://www.pnas.org/cgi/external_ref?access_num=Z22865&link_type=GEN) | Dermatopontin | 1.3 | 88.8 | 159.4 | 193 |
| [U59111](http://www.pnas.org/cgi/external_ref?access_num=U59111&link_type=GEN) | Dermatan sulfate proteoglycan 3 | 1 | 1.3 | 4.6 | 13.1 |

**Part III – Regulatory DNA sequences in the model (1000 bp upstream sequences from the transcription starting site)**

>COL2A1

gcagcttgcaccgtttagtctcggctccctccgtcctctgtggctctttcagtccccttcccagggcctgcccctgccccccacaccttcacacaggtctccttctgtgcagtaacacaccagctcttttcctggctgtcggctcaggccaacttcggcctgtgctccagaggaagccttcaacgcagagctggatgggggaggggtggagggcagtcgctgtgaacgtccaggtgggagtctggggaccaggtactgcagggaagggctaaaagataggtcggggtaacccttcagatctggctcagctagcctgtctccaagatttaggactctgaatctctgtgggctcctccctgtccccactcccaaacgcctgacgcggtgccccctcgccctccgctgctcctttctaccgctttccctcctccctcccatgtcttttccgtccttggtctagggctctcggcctgcgcctctgcaaacaccccctcccctccaactccggcagaactccgaggggaggggccggaggccacccttcccgcctgtggtcagaggggggcagcgccgcagccccgggtttggggggcaggggccatctctgcgccccgcccgatcaggccactcggcgcactaggggtggagggcgggaagcgtgactcccagagaggggggtccggcttgggcaggtgcgggcactggcagggcccaggcgggctccgggggcgggcggttcaggttacagcccagcggggggcagggggcggcccgcggtttgggcgagttcgccagcctcgaaaggggccgggcgcatataacgggcgccgcggcggggagaagacgcagagcgctgctgggctgccgggtctcccgcttccccctcctgctccaagggcctcctgcatgagggcgcggtagagacccggacccgcgccgtgctcctgccgtttcgctgcgctccgcccgggcccggctcagccaggccccgcggtgagcc

>COL3A1

tcaaagacctataaatggtatttgcccctctttcaaatatgacacaaaaattactgcttagatagatccagaaatacattggaaagttttgtggatgaggatctggaaagtatacacataaaagtctaaattagaaatatttatactaatctttagagtaatgcagaaaaaatgaccactttctctggtactcttttgctattctatgtgataaactctttctcttccagtttcctaaaaagttatgtcatgaaaaggctgaatttttctctagaaaaatctgctctgtctttcccaggcagcataaaaattggaaatctcacaagaaagcactctggcttgctaaagaaatttgtgagaaatggaaacagctatagataagtatctaacttttaggaagccattcaaacattgctgaaatactttgtcttttgatatttgcctgaaacttaacttctaggacccagggtgggtggatgagtcgaaggggcatacggtggcatttctttccgtgagtctcttacagtctcctatttaaattgagttaggattacttctggcaaaatcccaacataaaaatcttctaggaagatcagttctgtaaattagacatactagataaatgggcatcaagcagtttttcaaaattatgcagttgttaacttcataaggggaaataaaaatgtatgcatttacattgtatgtattaaaacaaggcagagcatttctatacgttcctaagttatacaaacatatatgtaagagtgaaatatgtaaaaaaacttttacataagcagatgcatacaaactccagatgtgctctttttcttactgtgggttgtgtcttctataagggaaaaagaaatatttatcatttcttttactgctgaggggatgggtgcggctctcatatttcagaaaggggctggaaagtgagggaagccaaactttttcctatttaaggccaaagcaaaggaatctcagtggctgagttttatga

>COL9A1

agcgatgctgataacatgcctctctctaggtttgtgctccttgtaatttgctctcaccttgttaacggaggataatatgttgcttctttccagatacagtgtcctctgctcaactctggtagtctcagagtttaagaataaacaacaagtagggggcttgatgttacattttatcaggatttctatccatgaggtagagagagggaatgtgtatttagtaataggcatggcactttgaaaaagttacttcattttctgcttcctcaactttcttatttggagaataagggtaacttcagtcttaccttataatgttgttgtgagaattaaatggcattaagctttgagcattttcaacagacggaggtgcataatgaagcgttagctatgaagacgatgacaaataatgattgttgggtgttagaccctctgcctttgatacctcatttaattctcaaaaccattgttttaatgtaagcattttcaatctgttttacagttagggcatctagttacagaaaggtgaagtaactctctcaaggacacacagctagtaagcttcagaataaacagggattgaaacttaggttgatcgggcaccaaggctcccacgagtttccacacctctgcctcccagtgggcacatttttactggaacctcagccctctgaaagcttccactgtattcctatagcagttctgaaagctgccattgtactcctatagcagatctaaaagcatctactgtgttcctatagcaccttgcctggatctttctggtgaatttccccacatcctgattttatttttttcttttcagcaaactttcccctgtaaatccctccttcagtataaccttgttagctttgaggacacacccctaggcctgggctcagagcgctgcttctccccacccctttcctttgcttcagtttaaagtgtcacgagatgcctctggttctctccctttgcttttagccctcaccgggggcaggaggga

>COL9A2

ggaaggctctctggagtcgggctatgctggacagcggggttcggaaactctggtgaataaagtggtagttgtttctatgaacatggagtgagaaggcccaatcctaaacacaccagtgcttaaggcagctgcagagaattaaaggcaatagaaaaaggggagctggatggcaggagcttggatggggaagacttcaagggcaggtgaagatgccaacagcttcctgcctggaggaccgaagaagggttgggcttcactgcagacctccaggagacagatttgcttgttgggacagagtagggaggtgaaagcgaggtgaggaagttcgggggtgggggaggtgcacacagctccactaaagagctggaagaggccgatggagtattcatgagacggctgatccaggaaggactgtggcatattaatatggaaaaggcctgtttgtttgtgcttggagaggaaggagccagaggagaggggagcaaggtgggcgggagctggggagagcctgcagcaagatctgcagagcccgaggtgctctcggcctgggccctggagaggacgtagggtaagtgatggggcacaggctgtgcgtcagggggaaggtgctggggcctgaaggtcccgaggggaacctcagacggccttatgccttctcacaaagaaactggaccatcatctccgacccgcaccccggtcgcccctcgaggacagagggtgggcgcaggaggctggaccgagcggggcggagctggatgcctggcgccggcctccctcccggcaaccccgccggtcctctcaggtgacagctcacgccggcccccgccccgccccccgcatattcaggagccccagcccaccctgcccgcgaacagccagcgctggaggagcgccgggagactctgccgtcggtgcgtgcgcggacacgcacccgtcccccttggtctcgccgccagccatggccgccgctacggcctccccccgcagcctccttgttctc

>COL9A3

attgcaacagtcatgagtccccacctgccgaaaggaagctcctgcagccccctacaacccccagggcagcctttctcgggaattttcaagattcctgggggaggggctggcatctgcgccttcactgagcctacaggcaactggaagctttgagtcccctagggcagcgactgccctggcagcctgaggcagagcttggccggacctggcgacccctgagcttctgggaaatggacatggccagcaccgccttcaggttcctgcccagggcacggttccttcaggccggggatcccggggaggggttcttcccctcggccagggtcatcgttttgcgatctctcctgggagtctgggtttggagtctggtcttagccggtagcaactgacgtggcctgaccacccggcccgtccaggtccacgggtgaggggccgcggtgggggtgctcccagcccagcaggcagcgctggacagtgaccccggagcgggaaccagggctgcgctgggcactgaccgggccctggtaccggggattcaccctccccggggtgttcctgggccttgggtcgcctgggtccgctccggcgcctggggaggggtctgcggcttcggaaactcgcgggtctcccctgcccctccctgaaggcggcccttcagcgccgcgcgcttccgcccccacactcgggttgaggagcaaggagagaaaagagcgtctttctctcttgctcaaagctgcgtgtgtgcaacgcgccagtcccaggataattttaactcgcggccggagagaacgcgccgcccgcccggcgtcttttttgttttcgcccaggcgggctgggcggcggcgcggggcgggtggaagcccccccgcccaggtgggcccggctgaatggggggcttgtgcaggcgggggcgggaaggggaaggggaaggggccgcccacctcccgccccgcccgcccgcgcgccgcccgccccgacgccgcagctcagactccgctca

>COL11A1

taaattactcaccctcttaatgtcagctcccataaattttttcctccttctccctcagagtacagttcaactcttttaagaggaaagccactgaatgaacctagtgctgaatttaaacgtttaagagataaacctgtcagtttcagatctccaaagaggacttccctacatgctctaggttttgacttctagggtccctccattctatcttcattcctttcttctaattattgggttaaaaaaataaaaataaaaacacttttcagagtaggcagcgcaatgagtccactggttccacgtctgccatacgaagcagatttttgcacaaggataaggttaccgctgccagcccaggactccagcggtgtggccgatcaggcgctggcctcctcccctctcccgccgaccagcgagaggctaacagacgggtgcaagtagacaagtagctgtttttattgaatacagaagctccttgaaaactccgtgtgctccggggctctcctgcaattcctttcattcccaaagtgctggagccaagcacgcgtcccccgtaactccctcccattttttctcggggatttggtaggcgaggagggaggagaggagtggggagatggggggtggttggtgggctgggcctgctcggagtcctcattcttgggctgagggaggcgggggcgggcttggggccgccccagagtcgtgtgattgggtctgaccctcagcctgcttgtcagtttcgccctgggagggggagctgggagcagggaggggagtgggcggaggagggggctgcccggagccactcgtccagcccactgacggcatgaagcctttaggggcacacagtactctcagcttgttggtggaagcccctcatctgccttcattctgaaggcagggcccggcagaggaaggatcagagggtcgcggccggagggtcccggccggtggggccaactcagagggagaggaaagggctagagacacgaagaacgca

>COL16A1

aaagcaggtccctccccagtgctggtgtaaaggcttgaaatccagtcccgtcccctgccagggatcacagtaataacgataatagcagcaaatatttactgagctcctctcatgtgccagaccgtgtgctaagcgcttacagcctttggcccatttcctgcttacagcaaaggtctatgaggcatatactctcacgatcccatcttccagatgagaaaacggagtatggttacacagcacatttggaaccgcaaagccagagcaggggccagggatttgagcccttccaacctcatggagtaaggaggaataaggggactccctcagtctcacttctctgggagtcaggccagagcagagggggttaatttagcacggaggagggagggggttcccaccctcctcctcccagtacagacagacctagggacagactgactgggaatgttaggcagaggaattggaaaaagctgaccaccgaggcttgggcggcagccacatcatgggtgccacgcacttgggaggagggtctctaccctgctccctccacccctgtcccctccccgcccccccaccccgcagccttctgggggcccagccagggacatggaaagggggagctcttcaaactccaggcctgactcttaaagggcccgcaggagcccccttccagggcagctctgggcacgtagggtcccgcctagctcgaggatcgctcacctggcggtgaggcaggaggggcgggcctgggagctcggccagacccggggcggggctgtgcaccccgcagtctagccgcctctggtgccgctggagccccctctaggccctcctcctcctcctccctggcgggccggtccagctgtgttccattagtgtccctggcaggcccccagccccctggaaccgcctctcttcccccacccggagtggccggcagtccccggcaggcgtgcgccgggcagcagacagccgggagagctcgagcgccaggaagctg

>COMP

gaaaattgagagtcccaggtctcaactgcccctcccctattttccattcatcatcataatcatcattactattaatcattaattaataattattaacttattacctccattgtgcaagggaggaattacgcctgggtaatttttgtacttttagtagagatggggtttcaccatgttagccaggctggtctcaaactcctgacctcaggtgatctgcccgccttggcttcccaaagtgctgggattacaggtgtgagccaccgcacccagcaacttacccagttttgaagcacttcaggaggagtggagggccagtcagtctgatccatagtgggtggacctattttttcagacgctggtgactctgtttcccgaagtgtgagctgagagcgtggccatggagcctgccttgtttggaactggaactcaggtttggcatacagcaagcactcaatcaatcaatcaatcaatgagctgaatgctatggctggatcctgtaatcccagttatgtggggagtatcgcttgaggccgggagtttgagactactagcctgcaggacatagccagacccggtctctaaaaataaaaataaaaataaaaataaattagctggacttggtggtgcgggcttgtagtcccagctacatgggagactgaagcaagaggatgcgatacagccacaccccgtcacacacacacacacacacacacacagacgcacacacacagtgaatgaaagtgggggcagtaccccctgactccctgccccaccagctctctccacagaccccgggactcagtttccccaccatgtcggattcagccgcgggcgacttcgcggggcattccgggcggggacttgaacgcaggggccagcgccatctgtttaccttgaggctggacgttgggcagggctgtggtgggccgtccctggggccggccgtgccttggggataaataggccccgcgggcctcgtgggcggtagaaagcgagcagc

>Dermatopontin

ttgacattctggaaaaggcaaaaccatagagacagaaagtaaatcagtggttaccaatggctgggtgtttgggaagatttgactgcaaagggatataaggggatgttttggattgatggaactgttctaagtcttgattagggtgctggctatgttactgtacattatgtattttaaaaggtgaattaattatatgtaaattataactcaatgaaaagaaaattcaatgtgagggcaaaaatactgaaggataaatagggggtttatttagattaaacccattacattttgagagcagagcacactttctagacttgactgggtccaatttcagcagcatggtagaatgcttaagtgtagaaatcatatccaacttatctccccatgggcccatagtacttccttatgcaacttatgagttctcagagtagccctgcaaactggtatacccgcgcttatactcaagggttaaaaagtgttggattgacaacttaaaaaaaaaaacaggcattcagccttacagaaatcctagatggctctgttacttcagtctatgatttcggtattttgaaatcactgtctgggtatttgtgtggcccggaaggctagtttggagaacaaatacaggggtttgctagtagcgggtagggtaggacagaaaggggtgaggtgctgaaaggtgagggacaaatatgctactaatcagtgtttttcaagtgaatatttctacagggtagagcagcagttggcttttataatctagatacaaagttgttctttctccagggagcttcttagacttacatgctgctggtatattcctagaattccaccttaaaacaagctgaatgtccttatggctgacatctcagcatggaaaagttttgcttaagcaatgacacactgcagttggatccttctcacccacatctggaagccacatgtcagcggcagctatataaaacggtcagaccagcaatcttagagtgacattgtttgc

>Matrilin3

ttaggatcgtgtgaggaggatcacttgtagagggaaaatagtgaaataggacgaccagataggctctgttggaactcgcgcccattgttttaagacgcagctcaaatgtcacctggttttagaagtcttcctggtctccgcaggcagtcagtatctctttcctaaatccaccacattcccgtagacagacaccacatttatgcaaatctccttctcacagacagtgagttccatgggggtcccctggcagcctccagccctgtaccatgctaactcttccggagaggaaatggggcctcgggagagcctttgaagcctgtgttggtacaggctgtccttccagctaacggggctctctggggaatgtatgtttgactttctattcccggtaatataagttcaaatgtttgtggatatttgaagctcggatgaaagcgtttctattcatgcccctagaatctgtgaggccttttctttctttttttttttttaaacaagagcccacagcaaaacttgggttacttggctgaggaaaggaatgtagagctaggagcagggtttcatcttcagccccagtcgggcaaaccagtggcagaagggaaagcagagcccaggggtccccatcccgtacctggggcgcaggccccgctggggtgggagggagagtggagggccgggcaggctgcagccgcggatggctgccccccctgccccggtgttactgggaggacggaggttgcgacagcagccgagcccacccagttgcagccggctcggggcttagggcaggggcgggaggtgaccaccgcgccgctggccgcctcgcccagacattccgtttctgccgctggaatgcgcggacaaggctccttgttggccttgggcggggttcgccgggccggcctggcgctcgaggccccggggcggggcggggcccgaggccgcgggacctttaaatccgagcctcgcgtgggctcctggcccccgacggacaccacca

>Aggrecan

gcttcatatgagatgtccttggtctctcccggggaatttgaagacccagagactcgcagctccccgtgagcctccccgctgacgccttacctccccctctccaccatcccctgccatcccggtgtacgccacccccaaaaaaactttcagccagctcctcggtgagggtcgttccaaatcgtggccgacagtagcgggctgcaccctccttagatcgcgtcgtggccagcctcagctgcagaaccccgccgggcgcccgggagccctgtcccgccgcggcggcctcagagtccgggcacttggggattctctgagggtgcagccctcctgggcccctccgactgcggagctgccgaccgcaatgcagatgcgggccctccagcccttctcgcgccgccttcctcccgccagccccggagctgccgggtccgcgccgccccggggagcgctcctctcccgccctgagcgcaggccggcttccccatcggcgcgccggtccggagccagggtccagcgcgctccagacgcctctgccttcccctccccctgtcggccgcccctcggtccctgggggtggggtttccctttgcgctcgccccctcccgcccccacccctcacgggccctcccctcccccgcccgtccctatgtatgtgtcacagcgcgccatgcccgcccgcccgcccacctacctccccgccgctccagagggggctcgcagagctgaggacgcgcgcagcgctgctcaaggtctctctctctcagcaccctcgccggccggcgtctgacgcgggtgccagggtctccgggcacctttcagtgtccattccctcagccagccaggactccgcaacccagcagttgccgctgcggccacagcccgaggggacctgcggacaggacgccggcaggaggaggggtgcgcagcgcccgcgcagagcgtctccctcgctacgcagcgagacccgggcctcccggccccaggagcccccagctgcctc

>Fibromodulin

actcatgctggcttgctctgttctctcccgcttcttgcagagacaaaatgcagtggacctccctcctgctgctggcagggctcttctccctctcccaggcccagtatgaagatgaccctcattggtggttccactacctccgcagccagcagtccacctactacgatccctatgacccttacccgtatgagacctacgagccttacccctatggggtggatgaagggccagcctacacctacggctctccatcccctccagatccccgcgactgcccccaggagtgcgactgcccacccaacttccccacggccatgtactgtgacaatcgcaacctcaagtacctgcccttcgttccctcccgcatgaagtatgtgtacttccagaacaaccagatcacctccatccaggaaggcgtctttgacaatgccacagggctgctctggattgctctccacggcaaccagatcaccagtgataaggtgggcaggaaggtcttctccaagctgaggcacctggagaggctgtacctggaccacaacaacctgacccggatgcccggtcccctgcctcgatccctgagagagctccatctcgaccacaaccagatctcacgggtccccaacaatgctctggaggggctggagaacctcacggccttgtacctccaacacaatgagatccaggaagtgggcagttccatgaggggcctccggtcactgatcttgctggacctgagttataaccaccttcggaaggtgcctgatgggctgccctcagctcttgagcagctgtacatggagcacaacaatgtctacaccgtccccgatagctacttccggggggcgcccaagctgctgtatgtgcggctgtcccacaacagtctaaccaacaatggcctggcctccaacaccttcaattccagcagcctccttgagctagacctctcctacaaccagctgcagaagatccccccagtcaacaccaacctggagaacct

>Osteomodulin

ggagtgcaaccaactgactaattttatctgaaaggatattagaagtcattctagtgggtacatctgccaaaaagaggagaaatgagatagctgataaaaatgatgataaaaatgaagatgtaagcctttgtatagaaaagtcgtatagttaatctgtgtaagcaatgacaatcaagtgcattttaaaaataaacaatgcgaaatgttatgtttctaatcctgatttcttgttttaacaaatagaagtactgatatgaacctaggtgttgaataaccaagggactcaagaaattttgagttccaggccaggcgtggtggctcatgcctgtaatcccagcaatttgggaggtggaggcgggaggatcacctgaggtcaggagttcaagaccagcctggccaacatggtgaaaccccatctctactaaaaatacaagaaattagccaggcatggtggctcatgcctgtaatcccagctacttgggaggctgagacaggagaattgcttgaacccgggaggtggaggttgtggtgagccgaggttgcaccactatactctggcctgggcagcaaagcaagactccatctcaaaaaattaaaaaagaaattttgagttccaccttaagttgctgagactctggagttccctgtcacagtgggccttaatttgaagaattctttggcatgaaaatgaaaataaaaacagtaatcagtacatgctgtttcatttagttttgtttgcctttcacattagaggaaattcttgccacacctcaaaccaaaattaaggggaaaagaaagaagtaagccacataaatctgaccaagaacacaagcatcttaatttgaatccacaaagtttcatgtaatgaaaagaaatacataattttaattcaacccgagtgttttccaagaagattgtatttgcttaaattgctacagtaattcaagagacagccctgtctggacacagagttactgtggatttttaagagactcagtta

>SialoProteinPrecursor

gatattcattaatttttgatttctcaagcagacttcgcaactggaggaagaataaaatgactagactaggagaatatgcaaactattaagctagatttccctttgtaaattaaaaaattagtactttagtttatcaatccattctttgtggtgttggtttcatgaatcatttcaaaaacaatggatcactcctgctagctctagtcattttgttattctcataggaaaaaaattaaatatgaaaatgaatagaaaagatatatatagaagcccaagaaaaaaatgagctgacctcacatgcacgacaggaagggccacataaatggacaatatacagagatttaatttacaaaacaaaatataaaatctgcctctcagtggtatgattctcaaaagttctaacttttatactcagcatcatgttttagcaactatatcttacaaagtctgaccgacttaatcatatcaactttaatttatgagtcaatgaagtatatttcaggaggaaacatcaaatgatattaaaatattgatggttcatctgctcctttcccttattatttagtttttctttctttttttagctaaactaatgtaaaattatatctaatgacagcaagctttcctttctttcgacatagtgaaaacttgtataattatgaaatttttaaaaggttaaagcctttgttatttattttaattcaaatccagtatattattatacatattcggagcccaaactattcatcttcatctaaaccttcaattaaattccacaatgcaaacctcttggctctagaatcacgtttcttgtttattcaactgagcctgtgtcttgaaaaagtgttgaagtttgggggttttctggtgagaatccacgttctgacatcaccttggtcgtgacagtgattggctgttggaaggcaaagaagagtttatagccagcaagagcaagtgaatgagtgagtgagagggcagaggaaatactcaatctgtgcca

>ECM2

ttgaacctgggaggcggaggtttcagtgtgctgagatcacgacattgcactctagcctgggcaacagagcaaaaactcccgtctcaaaaaaaaaaaaaaaatttaaaaaatagttctaaaattacctagtctaggataggcactgagttaggaccttcatatctgttattccttttattttcagaaaaatttgaaaggtatcaccctagttttacagaagagtgaactttggtcagaaacattaagtaacttactcaaggtccctcataaacagcagggttgcgcttggaatcctgatctgtctctaacttctcatttgcagataaagaaactgaggccaaagtacacaactaatttgccagcgtaacaggtaattaggagccacacagctccctttcacattttctttctattatttcatggttagaagttacataatttccactgatattaatacgtaaccatttttttgttgttgtcgttttgttagtattgtgtgtgagatcgaggtgggtgggcagatgagttaaatattattaatccaatttaaaatagattccagatatacacagcctttagctgacgagaatatcatccctataatctgataaagctcaagtgacatcatcctggctagtataaactttaacatttttaatgcgaatagtctatgaaaaccacagatttcttaatcaagctcatttttgtgtttccaactctgccttttcagcctgcaaacaaaatgctcttcccccacacccactgctgttttctacttttcttatgattaaaaaaaatatttcccttggtaaccattttccctggttactttcttggctgattttctgcacaaagaactgaaaggcatttatccccaagggaggcagttattttagattttactaagaagttcagcaaatacttttcaacattcccttctgtcctttctttgtttttaaagaaagctctgattttgtttcattttcagctggagacttaa

>AggrecanCoreProtein

gcttcatatgagatgtccttggtctctcccggggaatttgaagacccagagactcgcagctccccgtgagcctccccgctgacgccttacctccccctctccaccatcccctgccatcccggtgtacgccacccccaaaaaaactttcagccagctcctcggtgagggtcgttccaaatcgtggccgacagtagcgggctgcaccctccttagatcgcgtcgtggccagcctcagctgcagaaccccgccgggcgcccgggagccctgtcccgccgcggcggcctcagagtccgggcacttggggattctctgagggtgcagccctcctgggcccctccgactgcggagctgccgaccgcaatgcagatgcgggccctccagcccttctcgcgccgccttcctcccgccagccccggagctgccgggtccgcgccgccccggggagcgctcctctcccgccctgagcgcaggccggcttccccatcggcgcgccggtccggagccagggtccagcgcgctccagacgcctctgccttcccctccccctgtcggccgcccctcggtccctgggggtggggtttccctttgcgctcgccccctcccgcccccacccctcacgggccctcccctcccccgcccgtccctatgtatgtgtcacagcgcgccatgcccgcccgcccgcccacctacctccccgccgctccagagggggctcgcagagctgaggacgcgcgcagcgctgctcaaggtctctctctctcagcaccctcgccggccggcgtctgacgcgggtgccagggtctccgggcacctttcagtgtccattccctcagccagccaggactccgcaacccagcagttgccgctgcggccacagcccgaggggacctgcggacaggacgccggcaggaggaggggtgcgcagcgcccgcgcagagcgtctccctcgctacgcagcgagacccgggcctcccggccccaggagcccccagctgcctc

>ChondromodulinIprecursor

gagctctgcacaaagctagaatcgaacgcaatttaaggaagcctggatccaaatgaagtaccatgtatcctggaactggaagatggcatttccaaaagtgaaaattttatatacatacatacatacacacacacacacacacacacacacacatttgtgttattcctttggatacatcagtactctgtgtgtgtgtgtgtgtgtgtgtgtgtgctttgccattgttgcttttgtttgcacttatttaatttattcaaccaaccagcatgcacttttattctggtctccccaactccttccctccagccctgctctgcttagctctcacttttgctgcagttgtacccagaccctagaaaaactggtggcaaggagccatccctaagaccctgctctgttttccagtcccaggggccatccagcaatgccaggccagtgagaaggtggcctaacccttacactcttaggccagtgtcccctgtccacctgcaccactctgttgtcttctccttcaccttccatgagccatcttccccccacccccaccccaacacacacctacaaagaccctgcaggaaatggcctccagccagttgtcacagcagagcccgctgacattctaagacatcctccggtgcatggctaattgctctggcacttgagtatttctttgggtcccaggattcagcatcccgctctgagggatgcaaccacccccacacattcagagtcaccgggatgctgtttggggggccagtagacaggatagagtgaggaggaaagggggcatccgggagtgcaggacgagcttcccgcggcgggagagagaagggaggagagaggtgaggcgctggaaggggtggggaccgctgggctggcccaggcgggaccgtgcaccgtgtgtgcgcgcggcgttgaaatgccctgcacgtcggggcagcgggacagatcccagggtgcccagggagtctccaagtgcctcactcctcccgccgcaaac

>DermatanSulfateProteoglycan3

ttttgagggcagggaagatgggagcagaggcaggaaggggagtgagagaaccttaaagacaaaccttcttctctcccatcacttggcccaggagtaataccgctttcaaaattcaatacatctattcacttgcagacatgaaggaaaagattcctgaagcataagtgagaaccaaattcctagatttatctaaagcataccagaatacaagatgcaactttcacctttctctaggacttatacaatgtaaaattgaggtatggaaatttaaagcatattttttctcttcatatttgtattaaagttcaattttacaaaccatggaagttaacaagagcttaaaataaagctcctattctaaggaaaaaatgtgtaatttgatttatttgtctatgatagatacaataactttttttaaaaaaagccccttttctccccaaaacacgcagatcatatttatgcatcacttctggatgtaatttttttaaagttatgcaaaaggttcatttttccctgttttgctatcaaagcattccatgcaatcaaaataattgggatatctttttttacttacccaatattaaacaggtaaggtttctgcaacatttaccaaatttcacttaacattaatgaaaagtgaagaaagcaattcaagacttcaagttttgggaaaattatttcttattagaactaaaatatccatctatagtatcactgtatacatgaacttcatttcttaattggtgagtttgttacagatgaggtatgttaaatccaactttttccatttaaaaattttaaggtgatactttggcaattataactccattaacataatttcaagaactttgtatactatgtgatttggatcctactgcttagtttgggtgactataaaataatttgaggttttttaaagactgagaagtatttctcaatattaggttttgcaaacagaaagttgagtgttataaattccacctcccacagaccagaattc

>Prolyl4HydroxylaseAlpha2

gccaagggaggaggcgccgagctgaccgggcgacgccgcgggaggttctggaaacgccgggagctgcgagtgtccaggtgagcgccccgcccgctcagccgccagatcaaccttagcgctggggcgcgggctggggtcgccaggcggtgcgttctgcccgcgcggggctgagagttaggggccggggccggatccggggccgggggtcgcgccgctagccgccagcagcgcagtccgggccgccaccctgcaccctccgccctgtttctgcacccgtctgggttcttgtgccgccgcccgcaagccttcccgagctcagggtggtgaggtcagcggcgcccttcgtgcagttccctcggctgtcgggcggggctgggaacttggccgctcttccctgtcaggctcccgggaagtggcggcctgaccccgggctgccggctgttgggagcgggggcgcggcgtccgcctggccctgaggggcctcttcatattggctaagcccgttctgcaccctcccaagggctgggagtcctaggtcttgtccgggcagggtccagcttggagcccattagatgggccattggatcagaaagtcttttctcccccagacatccttgtggaaccagcgttgtttttccttggcagctgcggagacccgtgataattcgttaactaattcaacaaacgggacccttctgtgtgccagaaaccgcaagcagttgctaacccagtgggacaggcggattggaagagcgggaaggtcctggcccagagcagtgtggtgagcgctgtgctggaagggaatgcgggcagtgggtacttggtagagcactgactgcctccggccagaggacttcccggaggaggtgacccatgagctggagtggtcagaggaaggctggcaaaagggcatcgtggacagaggaacagcctatgtgagtgggagcagagaccttggccaatgccattccttatggccttgtagtggaagcaaggtgat

>Matrilin2Precursor

aggagaatggcgtgaatccgggaggcagagcttgcagtaagccaagattgcgccactgcactccagcctgggcgactccgtctcaaaaaacaaacaaacaaacaaaaacacaaaacctgaactgggtgcgttggctagtggtttgggaacagaattttggagctttataggagatgtcctggtctgttatacatccactcaccccagcctccaggaactggggagctggtagaggggggtgccggccactttgactgtcgctggccaatcctgtcttctgcatccctccaaggctccatggggacatccaaggtgaggttcctccattctgcaaagttgcaaatgtctctgctcataatttcaaaagtcttgtctgaactagactttccctgctccctcggggtaggaattttgtctcctctccatcctggcatctttacctcctccccatcccggggcctggtccccagccagtggacgctctgttaatatgaactggcactggtggggtgctctgggcactggcaagacagctcttggcgcttgtcatcaattagcccaattctcaccacttttttctccatccccgcgtcagtgggtgcgtcccgcaggtcatgaccccaagttctagatgtaagattcctgccatgtctcctttcccccagaagacccctccccgcactcccaacccgacaggcccctaaagctgcctgtgttccgtgcctgcggctccggacccctggagtctgatgcccgcctgggctcaggcacggacggagtggagccaggtggcagcaggcagggcgcgccgccagagtgcccagctcccggggctgcccacagggtggggccgccctcgccgccgctccctctccgcgccttccccatccccaaagaggacacccctccttccgcccggcccgctccccacccccgccgcggcaccgcctccccagccgcccgcccttgcccagcgcctcccaggcagccagcgagcgaa

>SOX4

ttttgtggctttctcttcccctttacagctttggctttaagaaagagctggggaacagattttgcaccagaggctgattctttattccaatgctgaaaagaactaataaaaataatcacttggcttttaagcagaaagcttccatttacagtatttcacccttaccccgccccgttccaagccccttgttatctaacataatggttagaatacatttccgtctctccccttaataatttcatgcattaacatcctcttcacacactatacacacacgcacgcgcgcacacacacgcgcgcgcacacacacagcaaaaggaaaaaagaggcagcagaaatctcagctgtacttctagcccttttaaaaagtttgctctgtaaattggaatgaggtcagatttggagcttctcattgcacgcggagattattattgcatcgggttccaagccaatgggaagcccgggggaggggtttggcatgaggaagcgttggttacagcagctgattggctgcagccaagactgtgaaaggataaagaggcgcgaggcggaattggggtctgctctaagctgcagcaagagaaactgtgtgtgaggggaagaggcctgtttcgctgtcgggtctctagttcttgcacgctctttaagagtctgcactggaggaactcctgccattaccagctcccttcttgcagaagggagggggaaacatacatttattcatgccagtctgttgcatgcaggctttttggcttcctaccttgcaacaaaataattgcaccaactccttagtgccgattccgcccacagagagtcctggagccacagtcttttttgctttgcattgtaggagagggactaagtgctagagactatgtcgctttcctgagctaccgagagcgctcgtgaactggaatcaactgcttcagggaaaaagaaaaaaaaaaaaaaaagacttgcctgggaggccgcgagaaacttgcattggaagcttcagcaa

>SOX5

tcaaccaccccggaggcagagatgagagcgcctcaaaacttttttcccctaccccaatctccgaggagtaggtgggtgcacagtgccggcggcggcggggacgcgcggcgcggagcctggctctgggaggcagtggccgcttcagcagccgaacctctcgcggctccagcctccgcgcccagcagcccgcggctcgcctcccggaaaggttccacggaccccgcagccccccgcgcgcgccctccctgccggcccgaccgcctgaccactggactagggggccgggaggggccaggtcctgggcgcagagcagcggctgaggctcaggcgccgggaagaagttgacaaagggacttgactaaacatgaatgcgtatttatagatgcgagctccatcgagatctagagatatcccgctctccctggctccttccactcctgccatttcatttttatcgctcctttaagtggagggcagataaaaaaaaaattcctgcacggtgtgggtaatcgccgcgttatttacaatctagttactctgcagcagacggggaagaaaactctggggctccttcccttcggatggatagagagggcggccgggcggcgagctgcaagaaggaggtcagggtgggcttgcgggagctcgggggcgacctggagggagcgggagcccccggcaccgcccccgcgccccccccccgcccccgccgcaccccccggggcgggcgccgggccgcggtgaccgccggctggtgcgctgccctcgcctcccccattgttcctactgtctttttgccttttgttatttgcactcgcattgtgttgtttgtctctgaagccgcctcatgaccagcaaagggatttcacctcgtccttgagagcaacacggggagcgcgagcgagcgggcgagcgagagacggggggagagagagggacacgcgcgcgcacacacacacacaccggcacacacgcacacacacagacacacacacacaga

>SOX9

aaccaagtgaccggcctgggcctcgcggcccgggacagccgcattggcaaacttctatctctcaaagccagagcagttagcaaactctcccccagacagggcgactcggctgacgtttttgacccggccaggaggcaaagaccaaaacgtcagagcagtagccctgttactgaggagcgtcggcagggtcgcgggtagagggggctggagaatgacttgtcagagctcaaggtcgatgtggcgcggggcggcctcgagagcgccgggctcctgcgtggccacggccgccgctgccaaccttcgcggggacttagctttgctttccattgactccctttgcaaaagcgcagcagaatcctgaccagccgcaccagccccggcgaacccgagcatgttaatctatttatatggattattacggaggaacagcgggcgttgagtcaccaaaacatttgcttcaaaagactatttctaagcacttttgcaggcaggcaggctcgctccaggcgcgtaaactcggctacgcattaagaagcggctgcttttcgaatactgcaaactccagctaagtccccggtgccgcggagagagcagtgaaaagaaatgtcggaggtgggggtagatcctagtctagacacacacacttgcgcgcacacacacacacacacacacaagattcgcgcggagaaggcactaaaattctggcattccgagagtacgacaaacttacacacttggaagtcccgggtcccccgccttccccgcagcaccccccgcccccccaccctaccgtccgccctttggctgcgatcccctcccctctcctcccctcccgcctcgtcacccagcccagtgccacaatcctcctccctccccaaaatcgggtccaatcagctgcctgccaaccctgggactgctgtgctgtgattggcgggtggctctaaggtgaggcggagtatttattaaagagaccctgggctgggagttggagagccgaaag

>HOXA7

tgtttccgaggacgcgttgtttgaggtcaccttaaggccagatcggtgtttcattgacgattctgttggttttatttcagtaagttctatatcaaaaggatctggcagctcctttccccgcccgggcgctataggtgttgtgacccttgggacagaagttcagagcgagggtgggggggggggaatacacatcgtgtatgaaaaccgactgcagattctagataatcttacgtattctcacatccttggcactacaggaagctagcttcttcccgcaaggtttactccagctctaagttagagacaaaggcccacttttacctcgaggtaaagtttacaagatttcagaacaggaagaaaatgaaggtttggttttgtttcgtttcttgaaaagaagttaatagtatgtctttctcctaggataaatagccatgcgtattttaaaaactatatataaaaggaatgtgtaagaaataacctcaactcaaattattgtggtagaagaagagggggggtcagacagtggaggggggcacagggaaacccagccacagactaaagagaaaggtaaaagaagcagtagaggagagaaacaaggacggggaaaaaaagaggagcggaaaagagggctgaggaggggaggggaggggaggagaggagggcagaagagaaggaacgagaacaagggaaaatcccccgggaacacagaaagatagagacccaggggactcccgcagagagggcctcttgggcttcagcgcagaggaaagtttcccgggcaccccctctcctcccctgccctccgccgcctgggccctgccctgcgtgcccccaggcccagcgcgcctccgggcgagtccccaggagcgcggcccaatggatcgctccgggcccgccccctcgcgcgctgattggccgccgccccgctggcctcgccttattagcaagttctctggggagccgcggtagggcccggagccggcgagtgctcccgggaact

>IHH

gcgaggccagggcggggtggggcgcgtccaggcggggagggcaaactcgggcagcgcagggggcgcagagggcagcgggcgggcggacgcgcggcggaggcgcgagcgggacgagggctggctagtgccggggccgcccgcccgagggggaggaggctgtgctgcccttgctgcaggttcgctgtcgagcgcacaggaggcagggacatgggtagggtgcggtctgcgcggggcccgagcccggatctcttccatttcccctctcactcggccccgggctgcgccgcagacggcagcagctcccgctccgcccgagccgcctgaccgccgggccggggtgctaaccgcggggccctgcagcccgccggcccggccagcccagcccagcccggcggcccgcagccccgccgcccgccgccccccgccgccgccgcgttgccaaaacaaacgggccggcctatttattggcggccggcgagcccggcagctcagagtcgaggcgccgagggggacagcgcgccgccaccagctcgggccctgggcccccgccccgcacttgagtcccgccggccctggccgcaccacgccgcccatggcgcccccgcctggagccccccggagccacccggacgcctgagcccccgcagcgctcccgtcgacgcgcctgcccatcagcccaccaggagacctcgcccgccgctcccccgggctccccggccatgtctcccgcccggctccggccccgactgcacttctgcctggtcctgttgctgctgctggtggtgccggcggcatggggctgcgggccgggtcgggtggtgggcagccgccggcgaccgccacgcaaactcgtgccgctcgcctacaagcagttcagccccaatgtgcccgagaagaccctgggcgccagcggacgctatgaaggcaagatcgctcgcagctccgagcgcttcaaggagctcacccccaattacaatccagacatcatcttcaaggacga

>Patched

cgccgggccgcccgggaagcctccgtccccgcggcggcggcggcggcggcggcaacatggcctcggctggtaacgccgccgagccccaggaccgcggcggcggcggcagcggctgtatcggtgccccgggacggccggctggaggcgggaggcgcagacggacgggggggctgcgccgtgctgccgcgccggaccgggactatctgcaccggcccagctactgcgacgccgccttcgctctggagcagatttccaaggtgcatttcagactctctcctcccactttctcttccctcctctaactctttgggatcgcccccgccacacacaaacacacacactctcttcctctctctctcacacacacacacacactcactcacacctctccaggaaaagcagcagacaaatggggattgaaaaattcaaaccctccctctggtcctgggaggaaagggctgtctgaggtccgcagggggtggaggtgtgtgtgtgtgcgtgtgtgtgtgtgtatacacacgccctccctggtgtgccttttccggagcactggaaagccgtccacggcggaccacctcaagggcggccgcggcactgtcctgccccgtgccccctgccctgaacttcttcctcctgcgcccctgcccctatttgcagcctaaactcctgtacggctgccacatttcttaacatcttggagggggaggcggagtggagagaggcggagagaggaaggggggagggagccgaaataaaggtggtttccttttttggcagccagttttggttttgttgagcatgaaatctctgctcccttaaaaaattattctcggaaaaagatatcccccccgttttccaggttttgagccgcctctccttagggcctggtcgggggaggaaaagttgtaaacaaattgccaccttaaattcgcggtgcgagtctgcggagctgccgggttcattgtgtttacgaggctcgctgaaatgtgtggaatccagg

>PTHrPR

cagctggaatgtgctgagtggcagctgccacgtgaatcattatacctcatcaggctgagcctaggacttccagacacaaaccagctcagactgggctcaggcagaagggcagcctccagccgggtcacagggcctgggggtgaggtttctgggatcttgcagggtctctgcctccctgtctgcccacatcttcatcctgagcaaccagccctggttctgaacacagctcgttctacagaaccacagagagaagtcatcctgtccacagccccctgcctttgagacctgtgacagcttaagcagaccctgggcctggaagggcctgggaggcagatagggagggcttcctggctgactcctcaccaggagcccacattccagtgggcaaggagtcagatatgaggtggctgtggggctgtaggggtcaggacacgggctggagtggaatgacggccccctccatctcttccgtctaggagccactgggtacccagtgctggcagatcaggctttcttgggcctcagttttcctgcctgtgacacaggcaagaaccacctccagcccccctctgatttctctgggtgccttaaacttgagtgaagtcactagtaggggctgtgttcatacctcgtggcagcctgatattcctgagatgtggaaaggaccagagggttagacagggacacagagactaagagagaggcatggcagggcaaggagaggactattgaggcacacacacgtgtctggcagcctgagtgggcccagttacctggcaggcagacccatgggtgctgaggggagggcccagccctgggcatctgaacaccggcacacttggatctgcctctgttgcctcctactcctgactaggagcctgggacaaagtcctgggccagtgtagggcttggtaaaaaataattaattttcttttcaagtggaagctctgactcaggtcttttcttgtccccagcagcactatccttgcttaggcctgag

>WNT11

tcctgatttcgccggacacccggcttgccgctccctagctgaaccccacctctgtgcctcagtttcctcatctgtgagatgaggcagcgatagtgcctatctcacggggtgctgggaggagcagatgcgatcgtgttagggaactttttttgtttgtctgtttgtttgagacggagtttcgctcttgtcgcccaggctagagtgcagtggcgcaatctccgctcgctgcaacctccgcctcccgggttcaagcggttctcctgcctcagcctcccgagtagctgggattacaggcgcccgccaccacgcccggctaattttttgtattcgtagtagagacggggttttgccatgttgggcaggctggtctcgagctcctgacctcaggtgacccgcccgcctcgacctcccaaagtgctgggattacaggcgtgagccaccctgcccggccagggaacgttctttataaaagcggaattgtgcccgaattgccccagcttactgaggaggtaaactgaaacacgtttgggaaggagaattgccgaggccctattaactaatgctcaatattgcagggataagccccagctccgggtcctcgactctgctgcctcgcctggactcctgattcctcattcctctcctttccgtcccaggttgcgtctgtccgggtttccccctctgtctccgtggctctcagccttgtcctctgagcgtccttctccctctcgctgtgtctgcccggtctctcctgggtctccggttctgccgcctcctgtccctccttccggctgccccggccagaggggctcgaaggcgcgttggggcccggggccacgctgggagacacgccttcggccgcgcctaattcgagccaggcgcgggacgcgtcccccggccgggcggagcgggaggggacgcccgccgcgctcggcctccccgggccccgacccctcctttgtaatttgaataaaacgcctcccccgcccgcgcgccgcct

>Frizzled2

aatgggctcctttgtgttggctacggggcaattacgcctcggagtcctggtccctttaagagtctcttaaagagacaggatcccatttttcagagggttttttaagggtgtgtggagggggagggcgagggatctacgtgtaaagtgaagtgaaactttcgcctgggctccagaaagagcggacagaaaaggtctttgtaaattgttagtttttttaaagtctgttttaatttttaaatttgagatttttcactcgatttgaaaaattggactaggaataggtggggttcgaggatcgggggcgggggcaagggtttaaagagtccccacccggcagagcacctcggcccgggaagaggagtgagccagggtcgagtagtgtcccccgatctcgccgcggcaactccggagaaatcccgggctgggcaaacagcccttaatataataatagcttgtctctttaaaaagcaaaagggggggaaagttttgttggcatctggtttctgatctcattatgttgtggtcgaccaatccagccctcgggcctggtcctcgggtttaaatctgattggccgagagcacattttgggttggtgcttagagctcgacggggcggtttcaaggatcccttttttggggggctgaggagagggcggggccgccagcgggggcccccttgaaaccgactaattgggatcggagaggccgaggtgagggctggaggaggcacaaagaagtcgctgggtgcagaagggaggggagagggggagctgagaggcgagaggaggaggaagaggagagagggcagcagcgcgcggtgtctccggctgctcagtccgaccgcggcaagcaagcgggcaggcgcaccgccccctcccccgcccggcctccccaactctgcggccgcgagtaaagtttgcaaagaggcgcgggaggcggcagccgcagcgaggaggcggcggggaagaagcgcagtctccgggttgggggcgggggcggg

>Forkhead

gctgccggccctggacttagtggacggagcgggagattcccgcccaggggaagaggttcccacggagggcatgcggtcgcgggtgcctttggattcgtgtgttcagggaggccagataatctcctccccgcgccggagcccctgttcctcatttctagcattttaaaaaaggtcaagaatacagtgaatccggctgggggctgccggctgggtgacgcgcctctggctagaccgaatggcacaggctgagcccgggggtgcggaccggtgggcacggagaagggtccctcggcgcggactcgactgcaggagccgcggcgccgggggtggggggcgtgtgccgctcgcgagggggccggaggacttgacagatcgcagcgaaagaaaacattaaaccaaaacaaaacccaccgattccccacgtcgttcagcaaagacatcgtgggtggagccaggagggagcgattgagtagaattcctcgcggccgcctccgcccgcccaccccgcgcccccggccctcggccccctgccctgccagcccgccgggctcggcccgcagcggaggtcagggagcagcgagcgcctcctcccacccgggcttcccgagtactcggctctgctgctccgtagtaaacaaagtgtcgccggcgcctccacgctggtttgcttcctagcaatcaaaacactgagaaggcgagagaatcacagaaacactcgagaattaccagaaaataatagagatccaaaaaaaaaaaaaggaggtgagtgtgtgaaagagaaaaacaccccactaccccccaccagcccaccgccgcctccccgtggaaaaccgggccccacccagcccggcgcccactggctgcccgggcggcggtgccgcatgcccattggccgcgcgggctgtcggtcaggggcgggccggcgcgcgcgccgccgcgggcggggggcggcggcagatcccgtaagtcgggcggcctggtagtcgcagcagccgctgcc

>hDIP

aagagcttttcaaactagcgctgttctttactgaatgccctctgcctcttgttagggcatttgtatttcttatttctctagaaatcagctccagtttggtttttatcgatctccagagccttctttggagatgccagttggtacaagaaagtgctgaactgtttacagtcccagcctaaggttcagggaggggatgtggtttaactgggccacaaagcccggtacaggactcatttgcatggcccctgacgccatgtgacgcagccggctcctcctatataaagaagcaggagccaaaatatctccgagtctgggttggactggcggccgtggagtttgtgacatacgaggtgacacccctcgagtcacttcccttcaactccagctggagcgcctgcttggctttgggttcgttctgcagccttcgccccgctcctagcctcagggccggactccagcgcagagcccagcccagcgcagcctgccagcagccacccagccgcccagccgcccagccccgcacgaaacccggccagagcttcctagcagcccgagccatgaacaccgaaatgtatcagacccccatggaggtggcggtctaccagctgcacaatttctccatctccttcttctcttctctgcttggaggggatgtggtttccgttaagctggacaacaggtaaccgctggccccagctccggcgcctcccgagccggggcgccggccggagctgggctaggagcccggtctctgagcgagctgtgtgccgccggtccctgttccccgcggtgcagcatcctgggaaggggcgggggggaggggacgcaggaaggggggggtcaccgccgctctgggatccgtcatcgaagcgtccctgcggccgctgcgccgtgaaaaccagctgctgcagccgcagaggcagccggaggcagaggggcggcgggcaggaccagacagggctgggcagggggctggccgagcgccgtgcgccgcttggga

>GOS8

agaatgactcatgaggaaatcctgactccttgaactcactgattctggtgcattacagctatgaaatctctatgtctgcagctttctctcccaccataaggaaatctgtgatttctattttggcaggagatgtgaccaaggactcctgaggaatgaccctatagacagacccagccacgtgcctagtagtgatcaaaaatgcaaacccaattaaagcactgtcttctgataaagtcactttctcttgttcaatccctaatgttcattctcaaagtgttccaagacgccccagcaagctgcagagcagatcccaccctgcccctgggcaatcaacgtttcaggttactccttccttaagttaagtggcagagagaaacgggcgagggtctccccattgcctcagttcacagaccagggggtgtcgaatgagtcctacagcaggacagcaaacaagaaatgaggcgcggggtcagggaacgcgctcaaaaaaggaagaaaaatcccactcttcattcgaaatcaggccactgcactccggcctcgtggcgggcgacctcctcctgccagggaatcgccgctctggcctcgggctggggagcccgtcagggtgggtgagggagcgccggcgcccccgccgggccgccgccaacttcggctccctccctccgtcgcaaagccctcgagcccgcccggccgccacgcttcagcaaaaggtcgtgcgcagcggcccacactgaagactctccatctgctcccacacctgccccagctggccgctgctatgtggcccgagtgcgcaagaagccggggccgcagacgtcagcagcgccccggcttcgagacccttcggcagcagccgtgactgccgccggcgggcgctgacccatccccgtgccagtctgcagccgaccaatccgcgtcctcttgaggcggggccggagccgcgaggccccgcccccaagccgaggcctcataaatgctgcgacgcacgcccagccgc

>BAPX1

aacagaaactacgagcatgatttatggaactgcattatcttggattttctccgcagatttgactcaacttagtatcagtgttctgttaaagcgctggaataagtggatacatctgcagaggtaagaaacgtggtttgggtcggatgagcctccttgttgctggagggtgcttcgaggaccaggcttaggagagaaggggttaagtcagaccctgtctttagcggcgcaacgtgcccagttttgtctgttaaagtcaacgtcgcccaactggagccagccaatggacggccgggagcgcccgggacagcccagtggaggggtgtggcctcaggctgtcactcagcagtgaggaatgacacccgggcgggaggggggtggtacagtggaacgcaagggaaggaaagagaaaagaaacagataaaaccagggcagggggtaaaaaagttccccgcgcctctcggaatccagcggccgcggccgcggctaccgccgccgccgccgggggtgggctccggaagagcctgcccagcagggaaggggaggacgggccaggagcgaaatcctgcaggcgaggaagggacagacgcgcaaagaaaaagtgactcgggctgtgcgccccggcggcgctcgggaggggactgctgccacggaggggcccccgccaatcctccttcacgtcccccgagggggcagaccccagcccgcccctaagggcgagaagcgatgagataatccagctgagctgcgaaaggagagcgtctggctctggattcggggggagggggtctccgggcggggtgggcctctaaggtgggggcgagggtcggcgctgatcctgggacggagtggggcgggagctgctgggccgcgcagcagtgggcggcggcggcctcgcacacccagctcactcgcgctgcggccgccggcgctctctgtcccgctcggagctgctcggcgccccagctgcccgccccgccggccgctcctgcccgcggcgcag

>Cadherin13

ttgctttggctatcaggaagctcttatccaaatcagagcaaatacattagaatttgggcttgtcatttcagtttgctgaacttttccttctggcccagattttctattttggttcataaattctattgcacaaatgtcctttattgtaaaacaccttaaattctttctaagggaaggctgcatggaaatgatacagtaaggtcttccctgcattttcttagattcctattagggaaggcagacctagatgtccctcttaccctcagtcccaaagccccccatttataaaatcccttaagcagtgactactgctgttctgagtacctggaggtagttcagagcttctgaaaggtaacatccatatacaaaaagaagttccttccgatccagatcccagctttggtgccagatgcacatttgaggagtaggtggctagtcagactctcacctgagcagttaataaaatctattgccccttaatggaattttttctgcaagctcgaattgatctgtcatctttgtgatttgtgagatggcagggaagcaccaaacaccatcatgacttgggccacagtggggggaaaaaaggaaaaaagaaaaaaaaaatccactgccaagccttgccaggcgtagaaagggctggaactgctggggccattttatctgatttattggaaatagagtggatcttattaacattttaataaagagaatctttttgcactaggctggaagtggccgccagtcccccgtgcaattccattctctggaaaagtggaatcagctggcattgcccagcgtgatttgtgaggctgagccccaacagtccaaagaagcaaatgggatgccacctccgcggggctcgctcctcgcgaggtgctcaccccgtatctgccatgcaaaacgagggagcgttaggaaggaatccgtcttgtaaagccattggtcctggtcatcagcctctacccaatgctttcgtgatgctgctgctgatctattt

>CD10

cgcttttactgcatgatgatacagctttattttgaacagtgaccaaatgtgttctttacaaagaatattgacgagttgtgtagtcttgaacacgttgcctgacttctctgtgccccagtttcctaattctttattctattctgtggcagtgtccactttttggtttaaagtttttaacacatctccagttgaggtgaaagattttaggaatacaggtatgtttttgcataccctgctttatacaggactacaagtatcaacttggtaactttttgtactattttgtttacttatcccccttactcccctcttaaatgggattgggcgtggttaggttggatattcaggtatcttttggtacagtgagtgaccattttcagaatgttgtctaaccagttaatagatactggtatcagtagtagtagtaattttgaactgtaaaaataaaggttgtttttatgactaaaataaactacttcatgtgttcattcgttttgctgtttatctatattaatatttcaaattattgcttagtcaaaacctcaaggtattagtcactggtttttgagcttataaaggaagccactggagcaagatataaaattaaccattgatgttagtccagagttcaaaagggtgagtttaaaactttcttaatgatggtacagcaattatttgcttataaactgctgaattaatcctacttgagtatttaaccaagtcttactgtctaattttgctgagattttcagtgatataccaaagcaaaacaaaacaaaacaacagcaacaaaaaacaaaaggttgtgactgagacctgtcaaattcatttggctctatatactttgcattcagcaacaaaattttgacttctcaacagcaaaaatgtatttctattttaaaataactgagccttttacttttatccagccacattaagcatttggacattttcttttttatttatttgtttttcattattagtattttcattttttgca

>CD24

ataagctaccatgccctgacttgcatcaattatttcacatgcattcttgaacgagctctaagcagtttacaaacctttatgttaatgactcacatacattaaaatagaaaataaatgaaatatcaacagaaaaatagtatgtcttctagaaatgaaagaagacttatcagttttagatgactgactcttctgtttacagatttttaaaaaatattttaagttctggggtagatgtgcagaacgtgcatgtttgttacataggtattcatgtgccgtggtggtttgctgcactcatcaacccgtcatctacattaggtatttttcctaatgctatccctcccctacccccgcacccccaaacagtgcctggtgtgtgatgttctcctccgtgtgtctatgtgttctcattgttcaactcccacttatgagtgagaacatgaggtgtttggttttccgttcttgccttagtttgctgagaatgatggtttccagtgtcgtctatgtccctgcaaaggacatgaactcatcctttttttatggctgcatagaattctgtgttgcgggtgggggcttcagggccgcggagggggtccccttagggtgggccgtctcctcctcgcccgccgcaggcaggagcgcgggggaccgaaaccgtgtagtttgcagcgtcaggcagcgggtccgcgcccagcgagcggctccccagctcctgggaggatgcggacccgggacgcccccgtgagctcactgcgcctggctgacacgaggcgctcacagaacaaagcaagggcttcggggagggcgcggccgcggggccgagcgcgcagatcgctccggaccgggacaccgcctgcgaggagcgccgaccagccgggaagggttcgcgctaggcggcgcccgggtcccgtcggccagggtctcgccggctcgccgcgctccccaccttgcctgcgcccgcccggagccagcggttctccaagcacccagcatcctgctagac

>CD39L3

gacatgcaggaaccataaggagggatggtcaaacagggcctgcttaggcctcttgctgggatcactccaagggtctctggggtcactcagaggagccgggaggctgcctgctgctgtacactgagacagactcctggggtgtttggatcacggcagcccacatgctgcctttttgttatcttttctgttttgcctcttccgggctgtacttgtcttgagtttttggccttatgacatccatattaaatggggtagggcagagcattgctcagtaaacagcaagaatggccctgggcatggccaaggcagttctgctaagtgtgaaagggaatgtgcctgccatactttgtgctttaggcccaagtgacctaggggccagcctgcctactggcctcactcccaacattacaggtgtttgactctactccaccccgtggagagacggagcacaggagccacccaccgggggaccacataaggtcatgcacacccacagagtgcccagtccctgcctggccttgtccccattttccctccacctagggttggggtgacaatggaggtaactagaagacagatcaaaaggttcttggtcttgacacagatgcaagaaggaggcagataatagacacctcccttccccctcgcccacataggctccggtgggttctcagggcaaggcctggctccctcctctccgacttcaggtacaggtgagtgtggagggaactgaaagaggaaaggtgaagtgctgtttggcttcgacctggagggggacagaaggctctctgggcaggcagggccggtcttttggcaagaggcgcgctctctctcccttccccttccatcgagggctgggtgtgaaggtgagagcaatcagaaaagaaggtgaagaggtacttggccttgacacggacggggggggcggtcccaggccgaggccgccccgcccccgccccgctatacccggcgccgcctcccggcgtctgagctgacacct

>CD44

tatgaagagatgtgaaaaaggaagtgtggaatgatggatgagaagttgtatgggaagatgaatagaagaataggtggttgaataaattaaaaggtgtgtggttggatgaatgaatgagtgggatgatagatggacctaagtggttagtggatggacaggaggatggatggatgtgagagccccagaaggacataaggaaagatgggtggatagatggatgggcggatggaaggatatttaggaggatgaatgagcatgtgtgtggagagaggtgcccattcacactggcttgaacacatgggttagctgagccaaatgccagccctatgacaggccatcagtagctttccctgagctgttctgccaagaagctaaaattcattcaagccatgtggacttgttattgaggggaaaaagaatgagctctccctctttccacttggaagattcaccaactccccacccctcactccccactgtgggcacggaggcactgcgccacccagggcaagacctcgccctctctccagctcctctcccaggatatccaacatcctgtgaaacccagagatcttgctccagccggattcagagaaatttagcgggaaaggagaggccaaaggctgaacccaatggtgcaaggttttacggttcggtcatcctctgtcctgacgccgcggggccagcgggagaagaaagccagtgcgtctctgggcgcaggggccagtggggctcggaggcacaggcaccccgcgacactccaggttccccgacccacgtccctggcagccccgattatttacagcctcagcagagcacggggcgggggcagaggggcccgcccgggagggctgctacttcttaaaacctctgcgggctgcttagtcacagccccccttgcttgggtgtgtccttcgctcgctccctccctccgtcttaggtcactgttttcaacctcgaataaaaactgcagccaacttccgaggcagcctcattgccc

>IntegrinAlpha3

aggacggactcatttctacattgacagtcttagcctttgataccagaagtcgcagcacttagggggaaaaatgctttttggtaattataattagcgacatttggttgttaccactgctaaatatgtctgtctttgctgaggagccaatctcagctcggatcagggtgagtggataggagaaataaatctcggcaaagacagcgaggggcctgagtgcgaggaagaactcccagagaccgccagccacctcttggggactcacagcgctgcggaggcgagtccagcattctagccagccgcgcacggacgtcggttcccagatgcgccaatgccaggtctctccagcccggctggccttttcagcagcagcagcagcagccgcaggaaagcggccagacaaatcccacccccggccccgcccgtcacatccatcttgctccccaggcgacgctttctcggagaagcgaggggcggggaggggaaatctaagcctttgggggttgccgacaggtgtttgggggagaaggcggagctggggtagggaggatgaggagggaggggcggtgctgcccctttaagaggaggcggccgagccgggatctttttctctttccccggaaggaaagcgcagcccgggctgggctcgcaaggtggggaggtgcgggactgggcgtggggaggcggggcgcgcgccgggggacccctccctcctgtcctccttgcggtcgaccggtgcgcttgccagatccgccgcgaagccgggatcgaaggcgacagcgcggccaagggggcgcggccgggacaagctgggggccggttgcccggggcagggacggcggcgacccggccgctggggaggcaggaagatagacccacggatcttaggaagggatccgagagcgcagctgtgaaactggctggggctgggggcacgaaaccgatcagcgctacggagcgcagcggccggcgggttccagtgtcctccggcggcgcggggagc

>IGF1

cagctggcttggaccatgttgccggccagtcacccagttgagggatttgaatgacatcataaccctcgagagggtattgctagccagctggtgttatttagaatacacaaaaatcagagaaagaaaacacactctggcacacagactccctctgtcatacacacacacacacacacacacacacacacacacacacacaggtttgagttatatggaaaattcaaacaacaggaaaattgtttgccccccaggtacccttctcccagagtggtggggtggggaggggacagtgacaggcagcctagtagaagaataaagaaaaatgttctatttcagttgggttttacagctcggcatagtctttgcctcatcgcaggagaaaaagtatgagacagtgccctaaagggaccaatccaatgctgcctgcccctccataggttctaggaaatgagatcacacctctcacttggcaactgggacaaggggtcacccgagtgctgtcttccaatctactttaccccagtcacttcagggttaaaattgtagagtttgctggagagggtcttatcgtcctttctttctttttttgttttaaataatgcatttgctctagaatctaaaattgctctcccatcccccatattcctttaatactggtaaggtgtattagcagacgtttgtgtcttcatgcccagcagaaagttaatcagaaaacagatccttattttctatggcagcataagtattttaatgtctgcgaaccctgtcactaacacacattcttttaagggaaaaaaatgcttctgtgctctagttttaaaatgcaaaggtatgatgttatttgtcaccatgcccaaaaaagtccttactcaataactttgccagaagagggagagagagagaaggcaaatgttcccccagctgtttcctgtctacagtgtctgtgttttgtagataaatgtgaggattttctctaaatccctcttctgtttgctaaatc

>IGFBP5

ctgccacccactccctccagggatgtggccctgcccaatagaaatacctctgcaaagtgacagagagggaagtgtcactccaatccacctttcttccctctaagaaccccacacacaaggacactttaggccaccatgcaaggttttcagacttagcatttcctccaaagtgtccatctctgcaactcactgctctcaataatgctgttgtatgaatgtccattcctgcagatacacaacacacacacatacacacacacacacagagagagagaaagagagagagaacctccctggccaacgcagctgaaggcaactggtcacctcttcatcatgagtactgaaaccccctgattaaatcctcttctccagacttttaggggagaaattcaattttttcttcttttttaaatttagctcaccagacatcactggccattctacactacctgtcccccaaacacacacacacacacaccccacatggaaacagacaccagcatacagtcaattgcagaagcttaggaagatttcttgggcacggtatatccagttggctaataagaaaatacgtctcccttcagcctgtgccttgactacttaaaggataggagggaaggggagacgaagttactctcctcattgtgttcaccctgctccgaagaactctgtcttccactggcccctccacctcctccccattctcggtagccccagcctgtcccccttgcccctttcttacattccggggggaggagggcgctgttcagaggggaggagggcgctgttcagggagcgaaggggagcccccttgtgtctagaaggcctctccccacccccaccccgtgtgagtttgtactgcaaagctccttggcatccttgcctgagttgggtgttgggaagctcaaattgcagctacaaactggctggcagccaggggccggctatttaaaagcgcctgctctcccggagccccgtagtctctttggaaacttctgca

>IGFBP6

cctgggcacaagacagtctgatgaatctagaaaaccccatggatgactgggagggaaggaaacggattctgggagaggagaggaatggaggagcgagcttacctgaaggacggggatggggaggggggtggggtctgctctctggccactgtccccaagagcatcccgaatacgagttcctttaattctctccctctctagtttcaacctgccaatttccccctttccctgcctcccagccagttctcaccctgcacacagccctctccaatccttccagactccgcatgcgcccaccacaatgctagggtctcctacggttcattcaagttgtatccctccctgcctcccggcccttcccccattggcttaccccagtctggttcccctcgggccccttttctccatagtcttacctcaagggtgctttctcttactcctttcccaattttgcccttcctctcctctgccctgttctacttctcccatcagcagtagaatttcccaagaccccgttatccaccctctccatcttttctctgtccctccacatttgcccactcctcttgtgatccacacacaccatctcttctcagttccctttcagcccccgtcttctcactccccaccgggtccctgcatgcccctcgtcgtcctacacatacacactaagtggattgcactcccgtcctccccttcttccgctcctccccaccctccagtccctccagccagctgtacagctcccaaattccgggccgagattcccgggaacaaagcaagaaaaatcagagcaatttgggaagtaaacagaaactggaaagggagagggaaagaagagtggggaggacccaggttgggggggccctctccagtcccacccagtttagggaatgcccctgccctctcccccaccccccttcacctggctcttaaagggcccggcccctggccggcggctacttaagacagaggggcggcggcgggcagcagctgcgct

>IL1R1

cttaggtcttacaagcttttcaaaggcccaaagaaatgattcagacaaaaaaaaaaattagactccaaaatactaaaagaaaactgcaaaaacaggtgaattaatggtgaaagatctgtaggacatagcagtaaggctaccacaacccagctttttgaggttgtcttaaattaatgtgatatggggtgtaggtatgttaacatgcttgataatggaagagtgaggatacctgcttcttaaacagggcctgctcatttttttctggcctctagtacatttctttctggcaaaactcaagctctgttttcacggagatgatgcactgagccatggcatccctaggtggtttgattcatttctgcaccccggtgtctagcaaagtgccacgtgcatgtgtttaatcaaaatagcttgtggttgtcaagctctttgtattcttccctccctccctgtgatttgatttcctgaaagtatcctttccccaaggattatggaacatacacttgttaaacactggacccattttcatgatcatttaaatgtcatgacctttctgacaccttgatatggtttggctctgtgtccccacccaaatctcatctaaattataatccccatgtgtccagggagggatctggtgggaggtgattagatcatgggggcagtttttcctatgctgttctcatgatagtgagtgaattctcatgagatctgatggtttaaaagtgtttggcacttccccccttgctctctctcctgctgtcatgtaagacgtgcctacttccacttccaccatgattgtaagtttcctgaggcttccccagccatgcggagctgtaagtcaattaaatcctctttcctttataaattacccagtctcagatatttctttatagcagcgtgaaaatagactaatacatgcctcttgagatcctcacagcaattggtgcctttagacctcttcattcacacttcactcatgtgttcttccttccccagg

>BMP2

gcgcagctcccgcccgctcggggatccccggcgagccgcgccgcgaagggggaggtgttcggccgcggccgggagggagccggcaggcggcgtcccctttaaaagccgcgagcgccgcgccacggcgccgccgccgccgtcgccgccgccggagtcctcgccccgccgcgctgcgcccggctcgcgctgcgctagtcgctccgcttcccacaccccgccggggactggcagccgccgccgcacatctgccgccacagcctccgccggctacccgaacgttctcggggccagcgccgagtggatcaccggggaccgcgaggcacccgcgcgccgcagaccccgcgcgggctggagcacccggcagagcgcgccacagcgccgtggcctctgctgcccgggctgcgccagagccgcggacgggcgcgcagagcgccggggactccggagccgatccctagcgccgcgatgcggagcacctactgcaggagatcgggggcctgggacgcgctggccgaggtgtgatcggaccccaggctagccacaaagggcacttggccccagggctaggagagcgaggggagagcacagccacccgcctcggcggcccgggactcggctcgactcgccggagaatgcgcccgaggacgacggggcgccagagccgcggtgctttcaactggcgagcgcgaatgggggtgcactggagtaaggcagagtgatgcgggggggcaactcgcctggcaccgagatcgccgccgtgcccttccctggacccggcgtcgcccaggatggctgccccgagccatgggccgcggcggagctagcgcggagcgcccgaccctcgacccccgagtcccggagccggccccgcgcggggccacgcgtccctcgggcgctggttcctaaggaggacgacagcaccagcttctcctttctcccttcccttccctgccccgcactcctccccctgctcgctgttgttgtgtgtcagcacttggct

>TGFb3

tttcaaaagggctcggcttttcctgtgcctgtttaaaacattaacatcgtgcagcaaaagaggctgcgtgcgctggtccctccctcccccaccccaggccagagacgtcatgggagggaggtataaaatttcagcagagagaaatagagaaagcagtgtgtgtgcatgtgtgtgtgtgtgagagagagagggagaggagcgagagggagagggagagggagagagagaaagggagggaagcagagagtcaagtccaagggaatgagcgagagaggcagagacaggggaagaggcgtgcgagagaaggaataacagctttccggagcaggcgtgccgtgaactggcttctattttattttatttttttctcctttttattttttaaagagaagcaggggacagaagcaatggccgaggcagaagacaagccgaggtgctggtgaccctgggcgtctgagtggatgattggggctgctgcgctcagaggcctgcctccctgccttccaatgcatataaccccacaccccagccaatgaagacgagaggcagcgtgaacaaagtcatttagaaagcccccgaggaagtgtaaacaaaagagaaagcatgaatggagtgcctgagagacaagtgtgtcctgtactgcccccacctttagctgggccagcaactgcccggccctgcttctccccacctactcactggtgatctttttttttttacttttttttcccttttcttttccattctcttttcttattttctttcaaggcaaggcaaggattttgattttgggacccagccatggtccttctgcttcttctttaaaatacccactttctccccatcgccaagcggcgtttggcaatatcagatatccactctatttatttttacctaaggaaaaactccagctcccttcccactcccagctgccttgccacccctcccagccctctgcttgccctccacctggcctgctgggagtcagagcccagcaaaa

>VEGF

tcagaaatagggggtccaggagcaaactccccccaccccctttccaaagcccattccctctttagccagagccggggtgtgcagacggcagtcactagggggcgctcggccaccacagggaagctgggtgaatggagcgagcagcgtcttcgagagtgaggacgtgtgtgtctgtgtgggtgagtgagtgtgtgcgtgtggggttgagggcgttggagcggggagaaggccaggggtcactccaggattccaatagatctgtgtgtccctctccccacccgtccctgtccggctctccgccttcccctgcccccttcaatattcctagcaaagagggaacggctctcaggccctgtccgcacgtaacctcactttcctgctccctcctcgccaatgccccgcgggcgcgtgtctctggacagagtttccgggggcggatgggtaattttcaggctgtgaaccttggtgggggtcgagcttccccttcattgcggcgggctgcgggccaggcttcactgagcgtccgcagagcccgggcccgagccgcgtgtggaagggctgaggctcgcctgtccccgccccccggggcgggccgggggcggggtcccggcggggcggagccatgcgccccccccttttttttttaaaagtcggctggtagcggggaggatcgcggaggcttggggcagccgggtagctcggaggtcgtggcgctgggggctagcaccagcgctctgtcgggaggcgcagcggttaggtggaccggtcagcggactcaccggccagggcgctcggtgctggaatttgatattcattgatccgggttttatccctcttcttttttcttaaacatttttttttaaaactgtattgtttctcgttttaatttatttttgcttgccattccccacttgaatcgggccgacggcttggggagattgctctacttccccaaatcactgtggattttggaaaccagcagaaagaggaaagaggtagc

>VEGFC

ctctttatcaccccctttttcaagtaaaccaacttcttgcagaagctgacaatgtgtctctttactctccacgaagattctggcccttctcttcacctgtcagaagtttaggattccaaagggatcattagcatccatcccaacagcctgcactgcatcctgagaactgcggttcttggatcatcaggcaactttcaactacacagaccaagggagagaggggacccctccgaggtcccatagggttctctgacatagtgatgaccttttcttggaacttttacaacccccaggacatttccaaactttgagcagggctctgggggccaggcgtgcgggagggaggacaagaactcgggagtggccgaggataaagcgggggctccctccaccccacggtgcccagtttctccccgctgcacgtggtccagggtggtcgcatcacctctaaagccggtcccgccaaccgccagccccgggactgaacttgcccctccggccgcccgctccccgcaggggacaggggcggggagggagagatccagaggggggccgggggaggtggggccgccggggaggaggcgagggaaacggggagctccagggagacggcttccgagggagagtgagaggggagggcagcccgggctcggcacgctccctccctcggccgctttctctcacataagcgcaggcagagggcgcgtcagtcatgccctgcccctgcgcccgccgccgccgccgccgccgctcagcccggcgcgctctggaggatcctgcgccgcggcgctcccgggccccgccgccgccagccgccccgccgccctcctcccgcccccggcaccgccgccagcgcccccgccgcagcgcccgcggcccggctcctctcacttcggggaaggggagggaggagggggacgagggctctggcgggtttggaggggctgaacatcgcggggtgttctggtgtcccccgccccgcctctccaaaaagctacac

>EGFRPrecursor

tgccaaaggaatatagctcaagttcctgcagcccaaaaaagctcagtttcttttggccaaagcttccgcgagtttccctggcatttctcctgcgggagctacaggggcagtgggacacttagcctctctaaaagcacctccacggctgtttgtgtcaagcctttattccaagagcttcacttttgcgaagtaatgtgcttcacacattggcttcaaagtacccatggctggttgcaataaacattaaggaggcctgtctctgcacccggagttgggtgccctcatttcagatgatttcgagggtgcttgacaagatctgaaggaccctcggactttagagcaccacctcggacgcctggcacccctgccgcgcgggcacggcgacctcctcagctgccaggccagcctctgatccccgagagggtcccgtagtgctgcaggggaggtggggacccgaataaaggagcagtttccccgtcggtgccattatccgacgctggctctaaggctcggccagtctgtctaaagctggtacaagtttgctttgtaaaacaaaagaagggaaagggggaaggggaccctggcacagatttggctcgacctggacataggctgggcctgcaagtccgcggggaccgggtccagaggggcagtgctgggaacgcccctctcggaaattaactcctcagggcacccgctcccctcccatgcgccgccccactcccgccggagactaggtcccgcgggggccaccgctgtccaccgcctccggcggccgctggccttgggtccccgctgctggttctcctccctcctcctcgcattctcctcctcctctgctcctcccgatccctcctccgccgcctggtccctcctcctcccgccctgcctccccgcgcctcggcccgcgcgagctagacgtccgggcagcccccggcgcagcgcggccgcagcagcctccgccccccgcacggtgtgagcgcccgacgcggccgaggcg

>FGF5

caggtctgggtaagctaaagcgagcagttagctaacccttcctgccacggggatcctggggcgaaagaggacaactgggcggtcagttacatgtgctggtgtccagccgctgcctctctttccccggtcggagcgcgggggtgcgtgtgccctcactctcacgaatacagacggagagcttcccagaaggaaaaacaaattcgtcataatgttcagcgcagccgagagtttccaacaagaaagcagctccagtcgtggcatgatgactcagggtggcgttttaaacgctgaataaacccattccttagctctttaataaatcgctaataacagcaaaaacagccttctccccgaaccgcgccacaccaaaggcttcccaagttgggctgcgaatccctgtttggttttccaacgccgaaagacgtagaagcagtgggatctcctggtgggacgcatttctccaagggctcccagagagaggacgagatagcagggtttacagggcaaagagaggagaacctggaaatcgtgtcctggggggcaagagtgcgttgcccagtcacaagtgtgctgtagaggtgggagaggacaaggacccatttccacatttcaaccctccgtttctatcgctgcccctggaggactcagctgctaacgccgagctcgtttccacgcggctctggtcctagtcggggaatccgggtggccgcgcgacgcgagagaactgctgggatgcgtggccctggtatgggcgcacccagcgtccggggcgcaggttctctcagcccagcttctcctcccactcactcgctcggatctcctccctccttcacatcccccgcccccgggaccgcgaggctccctccccgcaccggccagtgagtacacaaagccgcgggtgaggggaagcttcgcaggcgtgcacggagcagtgagatcactggcgttataaatatcccggtgccagcgcggagatccgctcgggtggcctctctcttcc

>FGFR2

tttccttatcagaaactgggaatagtactctgcagggtggttgggcagatgaaatagaatcacctatgtagtgtgaccctcccatagtagggcctccattaatattcagttttttcctctcatttctattgtttagaaggacagaaacatgcaatgtcctgcgaacatttccaagtggcttcctctcctccaaaagcttcagctgcctggcaaccctccccgcagtatcaagcaggcgcattttaaagtctctttagcgggagggcagggttagagccttcggtcatttgttttctaacttgacataaaacaacgtaacgcagtcgctcagcgagccgttggccagcctcgctcgcccggggaggagggaggaggtggcgcggggcgcgggaccaggccttattcggaggctgtcgctgcatccctacggccgcatcgcggccgagccctcgcctcccgggccggtgtctccggctgctcggaggcgtcgggagccgcgcgggactcggggcggcccgggcgcgcggcgctgattggcagagagggcgccgccgtccaggaaacggctcgggtttcagtgggggcgtgacccgccgcgaggaggcgggggcggcggcggcggcgcgggcggcggctggaggagagcgcggtggagagccgagcgggcgggcggcgggtgcggagcgggcgagggagcgcgcgcggccgccacaaagctcgggcgccgcggggctgcatgcggcgtacctggcccggcgcggcgactgctctccgggctggcgggggccggccgcgagccccgggggccccgaggccgcagcttgcctgcgcgctctgagccttcgcaactcgcgagcaaagtttggtggaggcaacgccaagcctgagtcctttcttcctctcgttccccaaatccgagggcagcccgcgggcgtcatgcccgcgctcctccgcagcctggggtacgcgtgaagcccgggaggcttggcgccggcgaaga

>RBP

tctcattccgtcattctcatcctttgattggctgctttgatcaaacgagtggaactctaacttcgaacagaaagagaaaaacagggtcagtaaattgtgccatcacacaggaaaatacctaaactagtcacactgttttgattcaattggctactgaagttatagaatgttgtttactcttctctcctttgtctactccccagccaacaaaacaaccgaccttagctgttttgaaaataaatgaaaattccaacatgggtttgaaataaaattgcatcataaacaatcggtaggtgtttttcaaagtggtttcagggaagtgccacggagtaagcaggcgaccaccgaggctgctaaaatatttcctgtcctgaccagggttgcgtttctggagaatatttaacagggagggttttaacgcttttaaagatgttgaaactaaagaacaaatattgaccagagggcaccacaacgctcctgaaagagagtaaaatacatcctttataaaatgaaaaactacttggatgaattattccaaaattcctgcacaagtggacctcagaaggcagacggaggcgccaatttggcatggccagggcctgggcactcacgcaccagggagcctcgcgggtccgctctcgctctgtgaggccacggtcttcccgccaggttgactcgagcctcctgccagagccactggccccggaggccaccctagaccgcagctggcggccgctggcacgagtgcagggtaactgagccagggccgctggcgcatttggcctggccgaggccaccccgcgcggccgctccactgtgcccgaggctgtcctggaggtgaggccggcccacagggaccctgcccgtgcccgggctccggtgagtcagggcgcgttatgcaagtgcccccggcgcctccccttcggtctttcaccccgcgcggttacgaaagcgcgaccccctccccccggcgctataaagcagcggggcggccgcggcg

>Smad3

cttgacggggatcagcaagcgcgaggccaagtgcgggccccaacttcctgggcccgggccttccgcgcgccccctctcgggctcgggtcctctccagggtgcactcgcgtccggacgacgcttacttgctgcgagtccaaccctctccggcccgcaactccaaccgagccgcccgagtgtggactccgagagcggacgccacgggccgcgctcagccctcccgcccgaggcccctctcgccgtgtttcccaggacttcctccccgcgcccgcccgacttcaggtcggggccgcgcagggtcccgcgcgctccagccaggactgccgccgcccgggtcgcccacgtgggcgtgctgggcgcggggtggtggccggccgggcgccctccccggagcagggggcgggcagggcgcccgcgggaggaggtggcggggcgcgggggaggaggcggggagccccggcggcgagggggcggtgacagcacttggaaaggaggctgcacgcggatttgcatgaaacacagactgggagcgggcgggagcgggagcgcggcgcacgccccgggccggcccagccagcgagcgagcgagcggcgagccgggaggaggagggtggcggggcggtgaggccgcagaggcggagggatctgcgcatcaaagctagcgaggcgagcgaagtttggccgggggttggactttccttcccggaggcggcacccaaacagctaccccgtgcggaaacccaaacttctgctgccacttggagtctcgcggccgccgcctccgccccgcgttcggggccttcccgaccctgcactgctgccgtccgcccgcccggccgctcttctcttcgccgtgggagccgctccgggcgcagggccgcgcgccgagccccgcaggctgcagcgccgcggcccggcccggcgccccggcaacttcgccgagagttgaggcgaagtttgggcgaccgcggcaggccccggccgagctcccctctgcgcc

>MMP13

ctgaggtgtaggaaccaaacagctcagcttttaagaaaatatctgagaatggaaagggtggaggtcataattagacagggacggtggtcaagaacattgagttttgagttattggcctaggttcaaatcctggtatcatttataaactatatgaccgtatacaagtatttaacttctcagtgtatcagattattcatatgtaaaatggaaaacaagctttctgacagcacctatgtggctgttgtaaggattaaatgagataatgccttttaaacctagaatgcagtaatccctgtttgtgctagccattacagttgttccgttccaatgtaattcttctacctctgtctgaatctgtagggaatttatgcaggactccatatttgtcgagaggtcatgccagtgtgtttaaattccaaccatggggctcaatcctgcggttgtgccgtagcacctccaagtcatcaagcttggttttggtccaatatcgtgaacttcaggtagacacaagacatctctctgtagttattctagagaaaatgcagtttataatttaccaggcagtagtaaacattctagaatcagtactaagtttctctttatggaagtaaacatgccatcttgatacacttattattcgaagaagcaaaagtagatacgttcttacagaaggcaaaaaaaaaaaaattttgctaagtgaagtaaaaaatgtactactctctgcttcttcccacagtatccataaatatgctgaggccgtttattttgccagatgggttttgagaccctgctgaaacaagagatgctctcatttatatttccctcaaattctaccacaaaccacactcgggagggaaaagaaaaagtcgccacgtaagcatgtttaccttcaagtgactgggaagtggaaacctatccataagtgatgactcaccattgcaggcctataaaagtaaaggtaatctctgcggaaagacaacagtccccaggcatcaccatt

>MMP14

ctttctcacggtcagctttatcctgccctctcccttccctacatacctccaccttttgttctgagtccagtaagtccctaaaggagattataactctttaagaattgcctcctttttcagtgtagaggtggaactaaaccccagagtcccaactcccaatcccttcttttacaataatttcacccaacttgatttgctttcccccagatcccacggccttgtacccttttcctcccagcgagttccaattaaaggtgcagccacattacaaatgacaataaaggaatcacctttgtctttctacaaaggttggagccacagatccggtatggttgtggggtttgttgttggtagttgggttatagtttggattttttagcagcagagggagggacatagacagttgtctacagggcccagaagataaccccctattagtaaactggccccttctccctctgcaggtctcatgggcatggaagaagaccccaccaccatcccacactctgagctcctcgttgcccctagccacatagcccccaataattcccaccctgaggtgagacaaatgctgaataccagaggaatcaagccactcagaatatgcttatagggacaaagtctcccacatcccgtcccctggatccccctacagccccctgctgtccatcgcggcctcaacccctgcagatggcagcctgcaccacaaaaaggcaacttagaggtgttttttttttttttccttccagttcttggttgtaattggattcaggctaaaacaaccacgtccccaaccaggaaaggagggcactggggcggggacggaggagaggctgtgggagaagggagggaccagaggagagagcgagagagggaaccagaccccagttcgccgactaagcagaagaaagatcaaaaaccggaaaagaggagaagagcaaacaggcactttgaggaacaatcccctttaactccaagccgacagcggtctaggaattca

>MMP16

cctctgattcccagcgcccctattcttccagggccgcttcctcaggaatgccgaccgctctccaggaccagggttgggccgctccccaggccccactagggtccgcagcctgagccccacccagctcatggctggagaggggcgcactcgtttttagatgcgaggcggcagggtgggcgggaagaaagaatggagccgcccaagttcctgggtgcctgcctcggggatgcgggatgggagatactgtcgaagagctgtgctcaaaccagggcccttgtcgcgggctggatacctccggtgcgccctaccgtagtggggaatcctttatgagccgtctcctttaagaagcgcgcgtgcgtgtgtgtcgggctgtgtgtatgcgcgcgtgtgtgtgtgtgtgtcgcggtgtgtctctgtgtgtgtgtgtgtgtgtgtgtgtgtgcgcgcgcgcgcgcgcgccaagggccccgagagccaggcagaccccgggaaggaggagttatgtagattacggatgaacattctggaggggagcgaggagaggagggaaggagaagcagaggcgagagagtgaggagcggcggaggcccttcccgcaggagctgggggcggcggcggcggcggaggaggaggaggagtaggcagtgcaggaggaggacgcacgggctggaggcggcggcggcggcggcggctgctgctgctgcggctgcgactaggctctttgagccggaaccgccggtgaacttaggcgccacgttcccggtgactgaccccggaggatggtgaacaggaactactgctgaccctgtcttcgccgctgcctctcgggggctgccgagcgcggggcccctgtctcctccccttgcccggctgtgggtgaacctgcaggctccttacccacccggaggacttttttttgaaaggaaacgagggagggagggagagggagagagggagaaaacgaaggggagctcgtccatccattgaagcacagttcact
